# Study of the Relationship Between Cyberbullying and Mental Health in Adolescents—A Systematic Review

**DOI:** 10.3390/children13030367

**Published:** 2026-03-04

**Authors:** Jorge Casaña Mohedo, María Teresa Murillo-Llorente, Marcelino Perez-Bermejo, María Ester Legidos-García, Miriam Martínez-Peris

**Affiliations:** 1 SONEV Research Group, School of Medicine and Health Sciences, Catholic University of Valencia, 46007 Valencia, Spain; jorge.casana@ucv.es (J.C.M.); mt.murillo@ucv.es (M.T.M.-L.); ester.legidos@ucv.es (M.E.L.-G.); miriam.martinez@ucv.es (M.M.-P.); 2 School of Medicine and Health Sciences, Catholic University of Valencia, 46007 Valencia, Spain

**Keywords:** adolescents, cyberbullying, mental health, depression, anxiety, systematic review, suicide ideation, prevention

## Abstract

**Highlights:**

**What are the main findings?**

- Cyberbullying victims experience significantly higher rates of depression (90%) and anxiety (87%) compared to aggressors, with effects often being more severe and prolonged than traditional bullying due to anonymity and lack of safe spaces.
- Vulnerable populations, specifically females and LGBTQ+ adolescents, show a higher susceptibility to victimization and severe emotional outcomes, such as suicidal ideation and post-traumatic stress symptoms.

**What are the implications of the main findings?**

- Effective prevention requires a multidimensional approach involving families, schools, and health professionals, moving beyond school-only interventions to include parental mediation and societal awareness.
- Interventions must prioritize emotional regulation training and the development of prosocial skills (empathy) for both victims and aggressors to mitigate long-term mental health deterioration.

**Abstract:**

Background: Cyberbullying has emerged as a major public health concern with profound psychological repercussions on the adolescent population. The shift toward virtual communication has fundamentally altered interpersonal dynamics, removing the spatio-temporal barriers of aggression and creating new challenges for mental health. Methods: A systematic review was conducted following PRISMA 2020 guidelines, including a comprehensive update executed in February 2026. Searches were performed across PubMed, EBSCO, Web of Science, and Scopus. The review included observational and experimental studies involving adolescents (aged 10–19 years) reporting clinical mental health outcomes. Methodological quality was assessed using the Joanna Briggs Institute (JBI) critical appraisal tools. Results: Forty-two high-quality articles were selected. Key findings include the following: Prevalence: A median cyber-victimization prevalence of 19.1% was identified, although significant methodological heterogeneity exists with ranges between 2.1% and 88.0%. Clinical Impact: Victims exhibited significantly elevated rates of depression (90%) and anxiety (87%) compared to uninvolved peers. Suicidality: Victimization is a critical risk factor, with suicide attempts reported in 19.0% of victims, compared to 3.0% in aggressors. Vulnerable Groups: Adolescents with autism spectrum disorder (ASD) demonstrated extreme vulnerability, with victimization rates between 64.1% and 68.9%. Additionally, females and LGBTQ+ youth showed a higher risk of symptom internalization and post-traumatic stress. The Role of the Bystander: Observers experienced fear, moral frustration, and helplessness, acting as either passive reinforcers or active upstanders depending on the school climate. Conclusions and Implications: Effective prevention requires a socio-ecological approach that transcends the classroom, integrating families, healthcare centers, and technological platforms. A transition toward modernized cyber-education is recommended, utilizing interactive tools and “serious games” to foster empathy.

## 1. Introduction

In the current digital era, the shift from face-to-face to virtual communication has fundamentally altered interpersonal dynamics and peer-to-peer aggression [1]. This evolution led Canadian educator Bill Belsey to coin the term cyberbullying in 1999, defining it as the deliberate and hostile use of information technologies to harm others [2]. While the concept emerged at the turn of the century, it gained systematic academic attention following the pioneering work of Smith et al. (2008), who categorized it as a virtual extension of traditional bullying characterized by perpetrator anonymity and a lack of spatio-temporal boundaries [3,4].

Globally, adolescent internet use has become the primary communication modality. According to the OECD (2019), over 97% of 15-year-olds access the internet daily in many developed countries, reflecting the near-universal digital connectivity of contemporary youth populations [5,6]. This hyper-connectivity correlates with rising victimization rates. The Cyberbullying Research Center (2025) reports a 58.2% lifetime prevalence in the United States, while the European Union Children’s Participation Platform (2025) estimates that 24% of European minors have experienced digital harassment [7,8,9]. Furthermore, the WHO (HBSC Study, 2024) indicates that one in six adolescents in the European region has been a victim of cyberbullying, highlighting the growing magnitude of this phenomenon across recent assessment cycles [10,11,12].

Cyberbullying encompasses diverse modalities, from direct harassment and social exclusion to more severe predatory behaviors such as cyberstalking and online sexual harassment [1,13,14]. These activities do not affect all groups equally; current evidence suggests a higher prevalence of cyberaggressors among boys and cybervictims among girls, with the latter showing greater susceptibility to depression [15,16,17]. Furthermore, emerging research highlights that neurodivergent youth, particularly those with autism spectrum disorder, face a disproportionately higher risk of victimization due to challenges in interpreting digital social cues [18,19,20,21].

While digital victimization often peaks during secondary education [22], recent evidence confirms its persistence into higher education, where university students frequently encounter their first episodes of harassment [15,17,23,24,25,26]. The multi-level health impact of this phenomenon—affecting physical, psychological, and social dimensions—can lead to severe outcomes such as anxiety, social isolation, and suicidal ideation [27,28,29,30]. To mitigate these risks, successful interventions have transitioned toward bystander empowerment, utilizing strategies such as the “Upstander” movement to reduce social reinforcement for aggressors [31,32].

Despite its prevalence, challenges remain in the systematic reporting of these events, including the rare but complex issue of false reporting in school settings [33,34]. Given the profound implications for public health and the academic environment, there is a critical need to synthesize the current evidence regarding the long-term repercussions of this phenomenon.

Beyond the identification of risks, current evidence highlights the efficacy of specific psychoeducational interventions. Programs such as Cyberprogram 2.0 [35] or the KiVa method [36] have demonstrated that fostering empathy and the ‘upstander’ role (active defense of the victim) significantly reduces cyber-aggression rates. These interventions often utilize positive behavioral slogans and peer-support networks, proving that digital citizenship education is a protective factor. Integrating these proactive strategies into school health protocols—often led by nursing professionals—is essential to mitigate the long-term psychological impact on the adolescent population [37,38].

Therefore, the main objective of this systematic review is to analyze the relationship between cyberbullying and its repercussions on the mental health of adolescents (10–19 years), specifically evaluating the prevalence, incidence, and associated epidemiological factors. Furthermore, this study aims to identify effective nursing-led interventions and protective factors within the family and school environment to mitigate psychiatric risks such as depression, anxiety, and suicidal ideation.

## 2. Materials and Methods

This systematic review was conducted following the PRISMA 2020 guidelines [39]. The protocol was registered on the Open Science Framework (OSF). While PROSPERO is a common choice, OSF was selected due to its flexibility in hosting open-access materials and detailed documentation of the review’s evolution, ensuring a transparent and reproducible process.

The review protocol was previously registered on the Open Science Framework (OSF) on 10 October 2024, with the identifier DOI: https://doi.org/10.17605/OSF.IO/EJK8T (accessed on 27 January 2026).

### 2.1. Research Question and Eligibility Criteria

The research question was formulated using the PEO framework (Population, Exposure, Outcome), which is specifically designed for systematic reviews of observational and epidemiological evidence. Based on this framework, the following research question was established: ‘What are the repercussions of cyberbullying (Exposure) on the mental health (Outcome) of adolescents aged 10 to 19 years (Population) in terms of prevalence, incidence, and associated epidemiological factors?’

For the selection of articles, the inclusion criteria considered were as follows: (a) experimental, cohort, or observational studies (including cross-sectional and case–control designs); (b) studies published between 2015 and 2025 to ensure the inclusion of the most recent evidence; (c) research focusing on populations in childhood and adolescence (defined according to the World Health Organization (WHO) criteria, encompassing individuals between 10 and 19 years of age. Studies focusing primarily on ‘young adults’ (over 19 years) or ‘early childhood’ (under 10 years) were excluded unless they provided disaggregated data specifically for the adolescent subgroup); and (d) studies providing specific data on mental health outcomes such as depression, anxiety, and suicidal behaviors.

Regarding language, no restrictions were applied to minimize publication bias. Conversely, the exclusion criteria encompassed letters to the editor, case reports, editorials, commentaries, grey literature, or any article that did not contribute to the fulfillment of the specific study objectives. This broader inclusion of observational designs was implemented to provide a more comprehensive epidemiological overview of the prevalence and psychosocial factors associated with the phenomenon.

### 2.2. Objectives

#### 2.2.1. General Objective

Analyze the relationship between cyberbullying and its repercussions on the mental health of adolescents, considering prevalence, incidence, and associated epidemiological factors through a systematic review of the scientific literature.

#### 2.2.2. Specific Objectives

- Identify the prevalence of cyberbullying in the adolescent population, considering sociodemographic and contextual variables (age, sex, geographic environment, and socioeconomic status).
- Explore the emotional and psychological consequences of cyberbullying in victims, including symptoms such as anxiety, depression, suicidal ideation, stress, and social isolation.
- Examine the psychosocial effects of cyberbullying on aggressors, especially in terms of risk behaviors, low empathy, and mental health problems.
- Analyze the role of bystanders in cyberbullying situations, as well as their influence on the perpetuation or mitigation of the phenomenon.
- Explore intervention proposals against cyberbullying, evaluating the role of educational institutions, families, and psychoeducational programs.
- Evaluate the epidemiological aggregates and risk/protective factors that influence the development of mental health disorders in the context of digital harassment.

### 2.3. Search Strategy and Sources Used

For the search of articles to be included in the current systematic review, the databases Scopus, EBSCO, PubMed, and Web of Science were used, with searches conducted between August and September 2024 in a first search and in February 2026 in a second search. While there is a significant overlap between Web of Science and Scopus, both databases were utilized to ensure maximum coverage of the literature. This dual-source approach was essential to capture high-impact journals in both the social sciences and clinical nursing fields that may be uniquely indexed in one of the two platforms, thereby minimizing selection bias.

The MeSH terms specified in Table 1 were used as keywords in the search.

The different search equations resulted from the combination of these terms with the Boolean operators “AND”, “OR”, and “NOT” (Table 2).

### 2.4. Study Selection

The literature search was conducted in two sequential phases to ensure the inclusion of the most contemporary evidence. The initial search was performed in August 2024, and a comprehensive systematic update was executed in February 2026. This updated search followed the same eligibility criteria and PEO framework, allowing for the integration of recent high-impact studies and responding to the rapid evolution of digital harassment trends.

Across both phases, the selection process was followed in four distinct phases. Initially, all records identified through the electronic searches were imported into reference management software (Zotero) to identify and remove duplicates. In the second phase, an initial screening of titles and abstracts was performed independently by two authors (JCM and MMP) to determine eligibility based on the predefined PEO criteria.

In the third phase, the remaining authors conducted a full-text review of the potentially relevant studies to ensure they met the inclusion criteria, particularly the reporting of specific mental health variables and adolescent populations. Any discrepancies during the selection process were resolved through consensus and discussion among the entire research team. Finally, the selected articles underwent a quality assessment using the JBI (Joanna Briggs Institute) Critical Appraisal Tools to evaluate methodological rigor and minimize bias [40]. The results of this process are documented in the PRISMA 2020 flow diagram.

### 2.5. Data Extraction

Data extraction was performed using a standardized template aligned with the PEO framework. For each study, the following information was systematically retrieved to populate the evidence tables: (a) Study Identification: primary author, year, and geographic location; (b) Methodology: study design, sample size, and age range; (c) Measurement Tools: specific scales used to assess cyberbullying and mental health variables (e.g., depression, anxiety, and suicidal ideation); and (d) Key Findings: primary results and clinical conclusions. For the 2026 update, additional attention was paid to intervention strategies and longitudinal follow-up periods to ensure consistency with the original data structure.

### 2.6. Synthesis Method

A narrative synthesis was employed to integrate findings from both the original 2024 search and the 2026 systematic update. Data were synthesized by comparing results across the categories declared in the extraction tables, allowing for a thematic grouping of evidence based on the type of psychological repercussion (e.g., suicidal behaviors, neurodiversity, and gender-based differences). This approach focused on identifying common patterns in the relationship between cyberbullying exposure and mental health deterioration, triangulating these findings with the methodological quality scores obtained through the JBI tools.

To address the heterogeneous nature of the data, we followed the SWiM (Synthesis Without Meta-analysis) reporting guidelines. This framework allows for a transparent and systematic grouping of diverse study designs and outcomes by categorizing evidence into thematic clusters, ensuring that the synthesis remains robust despite the variations in methodology and populations among the included studies.

### 2.7. Quality Assessment

The methodological quality of the included studies was assessed using the JBI Critical Appraisal Tools. This process was conducted independently by two reviewers (J.C.M. and M.M.P.). Studies were categorized as high, medium, or low quality based on their adherence to the checklist criteria. Only studies with a high-quality rating were prioritized for the primary evidence synthesis to minimize risk of bias.

## 3. Results

### 3.1. Flow Chart

**Figure 1:**
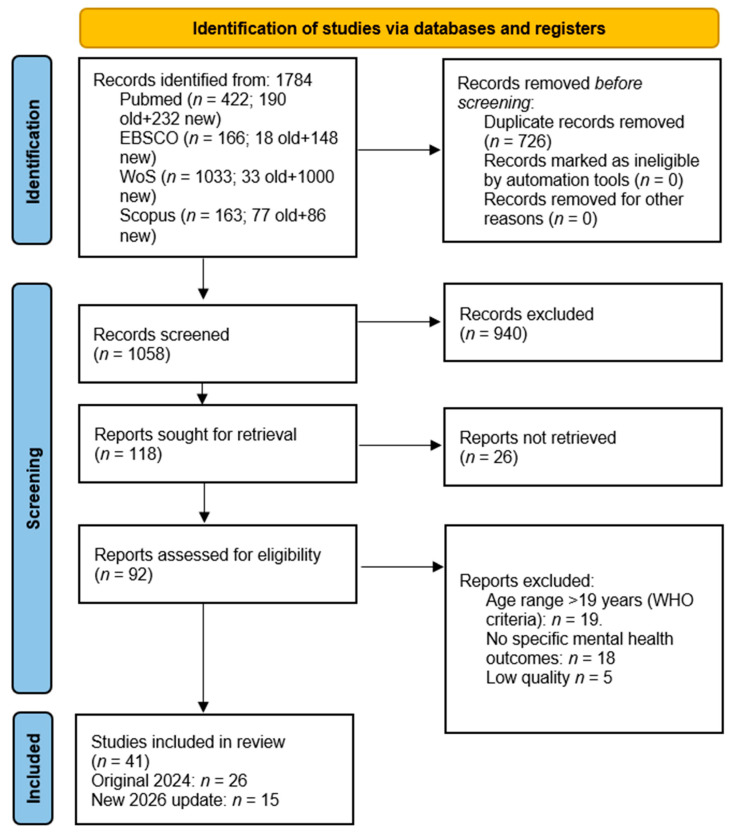
Flow diagram. Two searches are performed at different times: the original (old) search in 2024 and the new search in 2026 (new).

Below, in Figure 1, we can see the process of selecting the articles through the flow chart.

### 3.2. Articles Included in the Review

The selection of scientific evidence was carried out following the guidelines of the PRISMA 2020 statement. The initial search strategy in electronic databases identified a total of 1784 records: PubMed (*n* = 422), EBSCO (*n* = 166), Web of Science (*n* = 1033), and Scopus (*n* = 163). After removing 726 duplicates, 1058 titles and abstracts were screened, of which 940 were excluded for not meeting the primary inclusion criteria. Of the remaining 118 reports selected for retrieval, 25 could not be located, resulting in 93 articles evaluated in full text to determine their eligibility. During this phase, 51 studies were excluded for the following reasons: exceeding the age range of 19 years defined by the WHO criteria (*n* = 19), absence of specific mental health-related outcomes (*n* = 19), detection of duplicates in the full text (*n* = 8), and insufficient methodological quality after critical appraisal (*n* = 5). Finally, the corpus of the systematic review consisted of 42 scientific articles, integrating 27 studies from the original sample of 2024 and 15 new contributions identified in the 2026 update. The detailed synthesis of the included studies, the completed PRISMA 2020 Checklist, the quality assessment results, and the PRISMA flow diagram are provided as Supplementary Materials.

### 3.3. Characteristics of the Studies Included in the Review (Bibliometric Study)

**Figure 2:**
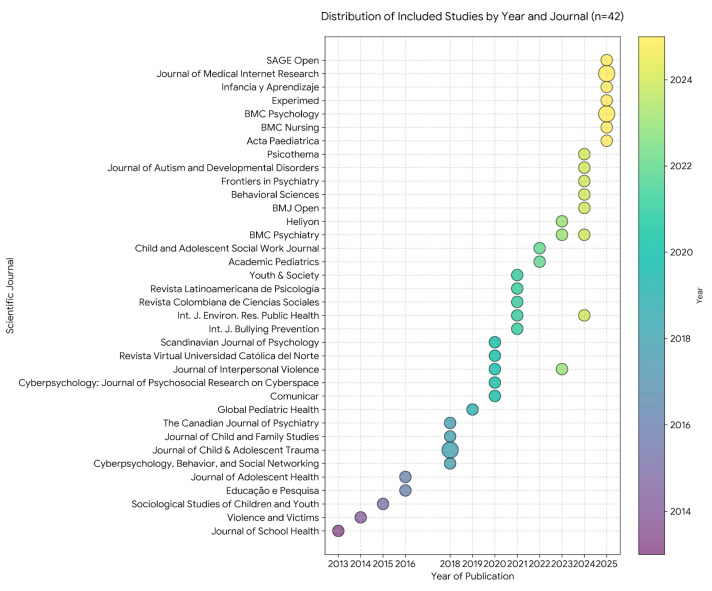
Comparison bubble plot representing the distribution of included studies (*n* = 42) by year of publication and scientific journal. The size of each circle is proportional to the number of included studies published in the corresponding journal and year.

The temporal distribution and source of the included studies (*n* = 42) are illustrated in Figure 2. A longitudinal analysis reveals a significant increase in scientific production regarding cyberbullying and adolescent mental health over the last decade, with a pronounced surge in publications during the 2024–2025 period. While early research (2013–2018) was distributed across general pediatric and school health journals, the 2026 update highlights a shift towards high-impact specialized journals in digital health (e.g., Journal of Medical Internet Research), psychiatric nursing (e.g., BMC Nursing), and clinical psychiatry (e.g., Frontiers in Psychiatry). This trend underscores the growing clinical concern and the necessity of nursing-led interventions to mitigate the long-term psychiatric comorbidities associated with digital victimization. The diversity of the 42 selected journals reflects the multidisciplinary nature of the phenomenon, integrating perspectives from psychology, social work, and evidence-based nursing care.

**Figure 3:**
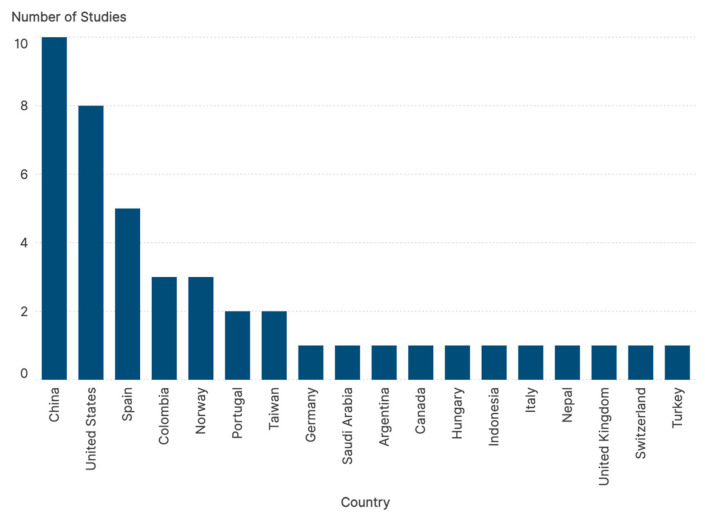
Global distribution of the included studies by country of origin.

The geographical distribution of the 42 included studies demonstrates the global relevance of adolescent cyberbullying as a public health priority. As shown in Figure 3, the evidence is distributed across several continents, with a predominant contribution from China and Taiwan (*n* = 8) and the United States (*n* = 7), followed by significant research clusters in Spain (*n* = 3), Saudi Arabia (*n* = 2), Turkey (*n* = 2), and Norway (*n* = 2). Other represented countries include the United Kingdom, Colombia, Brazil, Italy, and South Korea, among others. This international diversity ensures that the synthesized findings on psychiatric comorbidities and nursing interventions are representative of various cultural and healthcare systems, enhancing the generalizability of the results to different clinical settings.

### 3.4. Prevalence of Bullying and Cyberbullying

Significant variability in the representation of cyberbullying prevalence was observed among the selected studies (*n* = 42). This disparity often stems from the inclusion of different involved actors (victims, aggressors, and bully-victims), which tends to yield higher values than analyses focusing on a single role. According to the updated evidence, the prevalence of cyberbullying victimization ranges from a minimum of approximately 2.1% (specifically 2.0% in females and 2.3% in males) reported by Stea (2024) [41] in Norway to a maximum of 63.7% in the general adolescent population reported by Gianesini (2015) in Italy [42].

**Figure 4:**
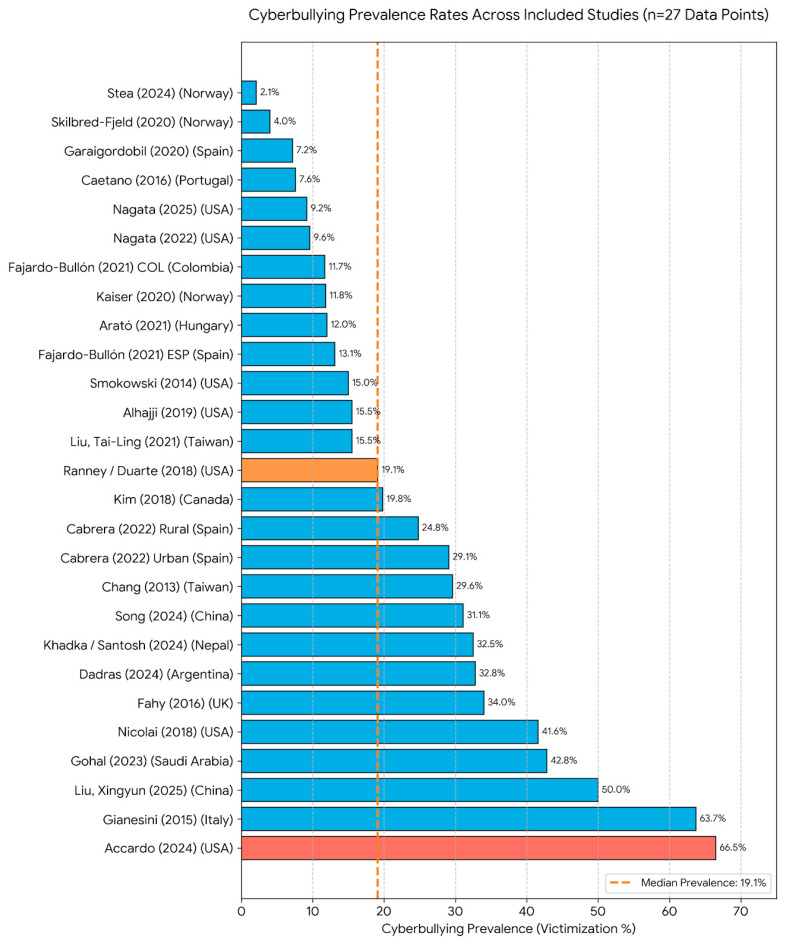
Cyberbullying prevalence rates across the selected studies (*n* = 27 data points). The dashed line indicates the median prevalence (19.1%). Studies correspond to references [21,41,42,43,44,45,46,47,48,49,50,51,52,53,54,55,56,57,58,59,60,61,62,63,64]. The orange bar highlights the median study [44] and the red bar indicates the clinical subpopulation (ASD) study [43].

Notably, when considering specific clinical subpopulations, Accardo (2025) reported significantly higher rates in the United States [43], ranging from 64.1% to 8.9% among youth with autism spectrum disorder (ASD), highlighting the increased vulnerability of neurodivergent adolescents. To address this heterogeneity, a median was calculated based on 27 distinct data points extracted from the corpus. Using the midpoint for studies reporting ranges, the median value was identified at 19.1% (represented by the study of Ranney and Duarte, 2018 [44]). Given the substantial gap between the mean and median in the previous literature, this median value of 19.1% is considered the most accurate reflection of the current global prevalence of cyberbullying (Figure 4).

The systematic review integrated data from a highly diverse global sample, representing 17 countries across five continents. The cumulative sample size exceeded 240,000 adolescents, with individual study populations ranging from small-scale clinical or qualitative cohorts (*n* = 31 [65]) to massive epidemiological surveys (*n* = 71,973 [43]). Geographically, the evidence was distributed among high-income and middle-income nations, including significant contributions from the United States and China (*n* = 7 studies) and Spain (*n* = 4 studies).

Regarding gender distribution, most studies maintained a balanced representation (approximately 50% male and female). However, specific research focused exclusively on female populations (*n* = 501 [45]) or showed a marked female predominance (83.8% [21]), reflecting targeted investigations into gender-specific vulnerabilities (Table 3).

The reported prevalence of cyberbullying victimization exhibited extreme heterogeneity. The lowest rates were observed in Norway, reporting approximately 2.1% [41]. Conversely, the highest rates in general adolescent populations were identified in Saudi Arabia (88.0% [54]) and Italy (67.7% [42]).

A critical finding from the 2026 update is the identification of heightened risk in clinical subpopulations. Notably, Accardo (2025) reported that between 64.1% and 68.9% of autistic youth in the United States experienced cyberbullying, a figure significantly higher than the median for neurotypical peers [43].

In the studies that analyzed both modalities (*n* = 16), the relationship between digital and traditional harassment (B) varied by context. In the United States, traditional bullying often surpassed cyberbullying (e.g., 31.0% vs. 15.0% [63]). However, in Taiwan, cyberbullying prevalence (29.6%) was more than double that of traditional bullying (13.3% [57]). Other regions, such as Argentina and Norway, showed more balanced rates between the two forms of victimization.

Rates of cyberbullying perpetration were generally lower than victimization rates, although they remained clinically significant. Reported figures ranged from 1.1% [60] to a peak of 55.6% [42]. In most jurisdictions, such as Portugal and Spain, the percentage of self-reported aggressors hovered between 3.9% and 13.4%, indicating a stable but persistent cohort of adolescents engaged in harmful digital behaviors.

### 3.5. Emotional Impact on Victims and Aggressors of Bullying and Cyberbullying

The psychological and clinical burden associated with cyberbullying involvement (both as a victim and as an aggressor) is characterized by a high prevalence of internalizing and externalizing emotional responses, as well as significant psychiatric morbidity Table 4.

Victims of cyberbullying reported a wide spectrum of emotional distress. The most prevalent immediate reactions were anger and rage, identified in 39.5–39.7% of cases, followed by significant levels of sadness (17.2–44.9%) and humiliation (23.6–25.9%). A notable proportion of victims (30.4%) exhibited indifference or no reaction, which may suggest a coping mechanism of emotional detachment or a state of learned helplessness. Furthermore, digital victimization was linked to feelings of insecurity (16.4–19.9%), fear (9.5–24.6%), and a specific desire for revenge in 35.8% of the sampled population.

In contrast, aggressors displayed a paradoxical emotional profile. While indifference was the most common response (45.6%), a substantial percentage reported positive affect associated with the harassment, including satisfaction (15.2–32.6%) and confidence or relief (8.9–26.5%). However, a non-negligible subset of aggressors also experienced guilt or regret (10.6–26.6%) and shame (8.9%), indicating a complex internal conflict regarding their behavior.

The clinical impact of cyberbullying is evidenced by alarming rates of psychiatric symptoms and self-destructive behaviors, particularly among victims:

Depression was identified in 69.6% of victims and 50.0% of aggressors, while anxiety scores were high and remarkably similar across roles (56.6% for victims vs. 56.9% for aggressors).

The most critical findings concern life-threatening behaviors. Self-harm was reported by 28.9% of victims and 15.2% of aggressors. Critically, 19.0% of victims reported having attempted suicide, a rate significantly higher than the 3.0% observed among aggressors, highlighting victimization as a major risk factor for suicidal behavior in the adolescent population.

### 3.6. Perceptions of Bullying and Cyberbullying

The synthesis of the included evidence reveals distinct patterns regarding the prevalence, emotional processing, and clinical outcomes of cyberbullying when disaggregated by sex.

Research indicates a higher probability of females becoming victims of cyberbullying, particularly during early and middle adolescence. Their experiences are predominantly characterized by indirect and relational harassment, such as the spread of rumors, social exclusion, and sexual harassment involving private photos or attacks on physical appearance (Table 5).

Conversely, males are more frequently identified as cyberaggressors and physical bullies. Their involvement often manifests through direct aggression, specifically insults and homophobic comments (e.g., “trash talk”), frequently occurring within online gaming environments. Furthermore, in certain contexts, males show a high prevalence in the dual role of bully–victim.

On the other hand, females experience a more profound negative emotional impact, characterized by the internalization of stress. This leads to higher reported levels of anxiety, depression, alienation, and psychosomatic symptoms. In some cohorts, this internalization is also associated with increased substance use.

Males generally report lower initial emotional reactivity or indifference. However, when they do experience distress from victimization, it often manifests as a desire for revenge, fear, or sadness. Unlike females, males tend to externalize stress, which is associated with increased aggression, conduct problems, physical fighting, and the carrying of weapons.

For females, virtual anonymity aligns with socialization practices that facilitate indirect aggression without the need for physical confrontation. For males, anonymity fosters “online disinhibition,” allowing for perpetration without physical force.

A significant finding in males is the avoidance of help-seeking behavior. Due to traditional gender roles and constructs of masculinity, victimized males are more likely to hide their experiences.

Significant differences emerge regarding suicidality. Females demonstrate significantly higher rates of suicidal ideation and are particularly vulnerable to interpersonal stress as a trigger for these thoughts following cyberbullying incidents. While males report lower levels of initial ideation, cyberbullying involvement is a strong predictor of suicide planning in this group. Most critically, rates of completed suicide remain much higher among male adolescents compared to females.

### 3.7. Contextual and Demographic Factors

Contextual factors play a fundamental role in understanding and addressing cyberbullying, as highlighted by several authors. On the one hand, Gomes [73] emphasizes that the school environment is crucial for preventing and mitigating bullying behaviors; for example, a supervised and safe environment can significantly reduce opportunities for cyberbullying situations to occur. Furthermore, the active presence of adults, such as teachers or technical staff, is essential to resolve technical or emotional difficulties during activities related to this topic. Likewise, Calpbinici & Tas Arslan point out that the family context also influences how adolescents face online conflicts, especially in terms of communication and emotional support [79]. In this sense, Kaiser [51] highlights that the anonymity provided by digital platforms exacerbates aggressive behaviors, as it diminishes the perception of responsibility for the acts committed.

On the other hand, Smokowski [63] underscores that the geographic context is also relevant, particularly in rural areas, where school problems and the sensation of insecurity may be more pronounced due to a lack of resources and community support.

Additionally, Fahy [58] notes that public policies and educational programs are fundamental to addressing cyberbullying comprehensively, not only by supporting victims but also by working with aggressors and bystanders.

Finally, Fajardo-Bullón highlights that the use of technology without adequate supervision increases the risk of victimization [56].

Therefore, it is evident that contextual factors are interrelated and must be addressed holistically to mitigate the impact of cyberbullying.

Regarding demographic factors, several studies agree that variables such as gender, age, socioeconomic status, and ethnic origin significantly influence cyberbullying dynamics. Fahy indicates that gender is a determining factor, as girls tend to report more victimization, while boys are more prone to be aggressors [58].

Furthermore, socioeconomic status also has a notable impact, as demonstrated by Smokowski, who used participation in free school lunch programs as an indicator of economic vulnerability [63]. This study revealed that two-thirds of the participants belonged to low-income households, suggesting that socioeconomic status influences exposure to cyberbullying.

On the other hand, Fajardo-Bullón highlights that ethnic origin also affects cyberbullying experiences, as students of Black Caribbean origin have a higher probability of dropping out of longitudinal studies compared to their White British peers [56]. Likewise, Gomes [73] points out that age is a key factor, as even a one-year difference can significantly influence how young people react emotionally to cyberbullying situations. This is because, during early adolescence, behavioral patterns change rapidly.

Moreover, Calpbinici underscores that cultural and ethnic differences affect how adolescents perceive their role within the community and how they decide to participate in virtual activities [79].

In summary, demographic factors interact with one another and condition both exposure to cyberbullying and the strategies employed to face it, reinforcing the need to consider these variables in future research.

### 3.8. Role of Bystanders

The synthesis of the included evidence emphasizes that bystanders [56,58,73] play a critical and complex role in the persistence or cessation of digital aggression. Far from being passive subjects, their behavior, emotional reactions, and decision-making processes directly influence the severity and evolution of the conflict.

The response of adolescents witnessing cyberbullying is not uniform. Four functional profiles were identified based on their interaction with the aggression [65]:

- Followers and Accomplices: Individuals who actively collaborate with the aggressor in executing the bullying.
- Passive Reinforcers: Subjects who indirectly validate the aggression through digital interactions (e.g., “liking” or sharing content), often without a direct link to the parties involved.
- Minimizers: Observers who strip the act of its violent nature, frequently categorizing it as a joke or a trivial interaction.
- Prosocial Bystanders: Those who actively intervene in defense of the victim by providing emotional support, confronting the aggressor, or seeking third-party mediation.

Exposure to cyberbullying generates significant consequences for the mental health of witnesses. Fear is the central emotion, driven by the dread of retaliation or becoming the next target of harassment [65]. Additionally, feelings of sadness, compassion, and indignation are reported, particularly when there is an affective bond with the victim. However, the coexistence of moral disapproval with the fear of acting often results in a state of helplessness and frustration [65].

Despite ethical disapproval of the act, prosocial intervention is often limited by several psychological and situational factors [65]:

- The fear of being socially labeled as suffering from reactive cyberbullying inhibits helpful behavior.
- The nature of the digital environment, with massive audiences, encourages the assumption that other participants or adults will intervene, thereby displacing individual responsibility.
- A significant proportion of bystanders report lacking the communication or coping strategies necessary to manage the conflict effectively.

However, defensive behavior shows marked differences based on gender and developmental stage: female adolescents tend to exhibit more prosocial responses and offer emotional support, generally in private. In contrast, males more frequently resort to avoidance or distraction strategies when faced with conflict [73].

Apart from that, greater maturity and emotional regulation are observed in students in higher grades, who show more prosocial responses. Younger adolescents exhibit greater vulnerability to peer pressure, which often leads them to suppress their emotions or ignore the problem to fit into the group [73].

### 3.9. Cyberbullying Prevention

The findings from the reviewed literature unanimously demonstrate that effective cyberbullying prevention and intervention require a systemic, cooperative, and multi-directional effort involving schools, families, clinical–therapeutic environments, society at large, and technology companies. It is imperative to apply a socio-ecological model that encompasses the complex interaction between individual factors (emotional regulation and digital literacy), relational factors (peer and parental influence), community factors (school policies), and societal factors (legal frameworks and media) [75,80].

At the personal level, studies identify the development of affective competencies—specifically emotional regulation and resilience—as a fundamental protective pillar against the impact of cyber-victimization, while also reducing the risk of perpetration [42,50]. Programs focused on emotional education and the strengthening of socio-emotional skills help adolescents process and cope constructively with their emotions, thereby increasing affective maturity [53,73]. Empirically, the development of optimism, self-mastery, and social competence acts as significant buffers (moderators) that reduce the onset of depressive symptoms and social anxiety following cyberbullying incidents. Furthermore, results highlight the necessity of empowering bystanders by fostering empathy and prosocial behavior, as witness intervention can halt the cycle of violence and minimize harm [49,53,73].

In the relational sphere, the family exerts a decisive protective role. Open family communication and a cohesive climate drastically mitigate the consequences of cyberbullying and reduce the likelihood of developing suicidal ideation [13]. Findings show that perceived strong parental support often has a protective effect superior to that of peer support, as it instills the security and self-efficacy necessary for adolescents to employ effective coping strategies against online aggression [71]. Parental interventions should focus on fostering democratic, warm, and accepting parenting styles, as excessively authoritarian (coercive) or neglectful styles increase vulnerability. Additionally, “e-Parenting” strategies (digital parental mediation) should instruct parents to identify early emotional symptoms and accompany their children’s digital navigation through assertive and empathetic dialogue rather than simple internet prohibition [75].

At the community level, educational centers must integrate cyberbullying prevention programs into their standard anti-bullying protocols, as both forms of violence often coexist [57]. Results support the efficacy of early implementation of multi-component, evidence-based interventions such as KiVa, ConRed, Safety.net, or Cyberprogram 2.0 [35,64,75]. It is crucial for schools to adapt interventions to their specific context; for instance, addressing different aggression dynamics in rural versus urban areas or designing focused support strategies for vulnerable groups, including females and sexual minorities (LGBTQ+ youth). Technological innovation is also applicable to prevention, with observed benefits in using “serious games” (such as Com@Viver or Cybereduca) and text messaging platforms to promote empathy and conflict resolution. Parallelly, ensuring continuous teacher training is essential to facilitate appropriate support within the classroom [44,73].

Once cyberbullying has materialized, clinical–therapeutic intervention takes center stage. Medical professionals, therapists, and mental health nurses must focus on early detection and providing holistic care aimed at improving self-esteem and equipping victims with healthy coping mechanisms [80]. Positive psychology-based interventions have proven useful for reconstructing moral cognition and reducing suicidal ideation [48]. At a systemic level, combating cyberbullying requires media campaigns to demystify aggression, digital literacy education from an early age, and the urgent update of national legal frameworks to guarantee privacy and penalize digital violence [75,77]. Finally, social media platforms hold a significant responsibility and can serve as active prevention tools by employing Online Ecological Recognition (OER) or algorithms to detect cyberattacks, block harmful content, and direct affected adolescents toward immediate psychological support interventions [48,75].

### 3.10. Intervention in Cases of Cyberbullying

The synthesis of the included evidence indicates that addressing established cyberbullying requires a comprehensive clinical, educational, and psychosocial response. Findings suggest that effective intervention must transcend punitive measures against the aggressor, focusing instead on a systemic process designed to repair harm, restructure relational dynamics, and involve multiple social stakeholders.

The critical first step in intervention is standardized detection and assessment. Research highlights that once cyberbullying is confirmed, schools should activate structured anti-bullying protocols consisting of three phases:

(1) Diagnosis and assessment through interviews with all involved parties (victims, aggressors, families, and peers);
(2) Design and implementation of an action plan (victim support, aggressor monitoring, and peer sensitization);
(3) Long-term evaluation and follow-up [75].

However, practical implementation faces significant barriers. Mental health nursing professionals report obstacles during assessment, such as adolescent isolation and refusal to communicate, a lack of rapid and precise assessment tools, and substantial difficulty in ensuring aggressor participation in therapeutic processes [65].

When cyberbullying leads to clinical symptomatology, intervention must shift to the therapeutic domain. The evidence demonstrates the efficacy of a structured approach based on three technical axes [75]:

- Behavioral Techniques: Social skills training, assertiveness, and self-advocacy.
- Cognitive Techniques: Cognitive restructuring to challenge irrational beliefs and self-control training to mitigate anger.
- Emotional Techniques: Use of play and dramatization to facilitate the expression of feelings.

Furthermore, mental health nursing emphasizes a “holistic care” model focused on enhancing adaptive coping, anger management, and resilience. From a psychiatric and transdiagnostic perspective, interventions should specifically target the mitigation of “thwarted belongingness” and the reconstruction of the victim’s belief in a “just world,” as these are key factors in de-escalating suicide risk following digital aggression [75,80].

On the other side, the literature reflects that intervening solely within the victim-aggressor dyad is insufficient; bystanders are central to conflict resolution [73]. Intervention should aim to transform passivity—often driven by fear of retaliation—into active prosocial behavior. In this context, technological interventions based on “serious games” (such as the Com@Viver project) have proven to be valuable tools [73]. By simulating real social media scenarios, these tools increase both affective and cognitive empathy, helping adolescents correctly interpret the severity of attacks and react by supporting the victim. From a social work perspective, emphasis is placed on classroom community cohesion to dismantle “moral disengagement” [55].

Likewise, family involvement is an indispensable moderating pillar once victimization has occurred. Studies show that establishing open, respectful, and empathetic family communication significantly buffers the direct effect of cyberbullying on suicidal ideation [74]. Furthermore, it is essential to adapt interventions to the cultural context: while in some societies (e.g., the United States) peer support is critical for recovery, in others (e.g., China), perceived parental support has a significantly stronger effect in mitigating emotional distress and self-harm [71].

Finally, at a systemic level, pediatricians and mental health professionals must create clinical environments of trust to facilitate the disclosure of abuse, which is often masked by psychosomatic complaints [54,60]. Current research outlines 21st-century intervention challenges focused on technology itself: utilizing social media platforms for personalized positive psychology interventions, promoting safe online support groups, and demanding the development of Artificial Intelligence algorithms for real-time detection and blocking of threats, supported by rigorous legal regulations that hold technology companies accountable for impacts on youth mental health [35,48].

## 4. Discussion

The results of this systematic review, updated to February 2026, provide a comprehensive and contemporary overview of the relationship between cyberbullying and adolescent mental health. By synthesizing evidence from 42 high-quality studies involving over 240,000 adolescents, several critical themes emerge regarding prevalence, psychiatric morbidity, and the evolution of digital aggression in the post-pandemic era.

### 4.1. Prevalence Heterogeneity and Methodological Implications

A central finding is the significant variability in prevalence rates, ranging from 2.1% to 88.0%. This heterogeneity is largely hypothesized to stem from the lack of standardized metrics and varying operational definitions of cyberbullying across different geographic contexts. To address this, our calculation of a median prevalence of 19.1% serves as a robust epidemiological benchmark, offering a more stable representation than a simple mean, which is often skewed by clinical outliers [41,42,43,66].

Notably, the identified “dip” in publications during 2022 reflects a transitional phase post-COVID-19 [17,76]. As adolescents returned to in-person interactions, digital aggression strategies became more specialized and shifted toward private, encrypted environments, temporarily affecting visibility in traditional reporting systems. However, the subsequent surge in research during 2024–2025 confirms that the phenomenon has not only persisted but has intensified [42].

### 4.2. Clinical Impact and Psychiatric Morbidity

The psychiatric burden associated with cyberbullying is alarming, with certain cohorts reporting depression and anxiety rates as high as 90% and 87%, respectively [43,52]. Clinically, it is essential to distinguish between adjustment disorder-related distress and syndromic depression [36,52]. While initial reactions may be reactive, the 24/7 nature of digital harassment prevents the resolution of stressors, often facilitating the transition to chronic clinical syndromes [13,49,60].

Within this clinical spectrum, the findings highlight social isolation as a critical consequence of cyber-victimization. It is essential to distinguish between cyber social isolation, characterized by digital ostracism and exclusion from peer networks, and traditional societal isolation, which manifests as physical withdrawal from school and family environments. Our synthesis suggests a synergistic effect: the ‘digital ghosting’ experienced online often exacerbates social anxiety, leading adolescents to seek refuge in physical isolation, thereby creating a cycle that complicates nursing-led psychological recovery [49,54,75].

The findings regarding suicidality are particularly critical for nursing practice [80]. With 19.0% of victims reporting suicide attempts, cyberbullying must be recognized as a primary risk factor in adolescent psychiatric triage [52]. This risk is moderated by gender and neurodiversity; for instance, autistic youth report prevalence rates near 69%, highlighting a heightened vulnerability due to challenges in interpreting social digital cues [43,47,59].

### 4.3. Modernized Interventions and the Role of Nursing

The evidence unanimously supports a move away from traditional, unidirectional educational models. Effective prevention now relies on modernized cyber education, utilizing “serious games” and interactive platforms to foster empathy and conflict resolution [36,73]. Furthermore, the transformation of bystanders from “passive reinforcers” to “active defenders” (upstanders) is essential to dismantle the cycle of violence [65,73].

From a nursing perspective, these findings advocate for the integration of school mental health nurses in multidisciplinary prevention programs [80]. Nurses are uniquely positioned to provide holistic care, focusing on emotional regulation and resilience, while addressing the “thwarted belongingness” that often precedes suicidal ideation in victims [68].

### 4.4. Limitations of the Study

It is imperative to acknowledge several limitations within this systematic review that may influence the interpretation of the findings.

First, despite adhering to the PRISMA 2020 guidelines, the search strategy was restricted to four major databases: Scopus, EBSCO, PubMed, and Web of Science. While these sources are comprehensive and capture high-impact journals in clinical nursing and social sciences, it is possible that relevant studies indexed in regional databases or grey literature were excluded. Specifically, a lack of high-quality evidence meeting the Joanna Briggs Institute (JBI) criteria was noted for several developing nations, such as India, Sri Lanka, Bangladesh, and Pakistan, which limits the generalizability of the results to these specific cultural contexts.

Second, significant methodological heterogeneity was observed, with cyberbullying prevalence rates ranging drastically from 2.1% to 88.0%. This variability suggests a lack of standardization in measurement instruments and operational definitions. To mitigate this, we utilized a median prevalence (19.1%) to provide a more stable epidemiological benchmark and reduce the skewing effect of extreme outliers.

Third, the reliance on self-reported measures may introduce response bias, particularly regarding sensitive mental health issues. For instance, the high prevalence of depression reported (up to 90% in some cohorts) primarily reflects scores from screening instruments rather than formal syndromic clinical diagnoses. This distinction is critical, as these figures likely encompass both adjustment disorder-related distress and chronic clinical syndromes triggered by the continuous nature of digital harassment.

Finally, while the 2022 period showed a slight dip in publications, the execution of a comprehensive 2026 update allowed us to capture the subsequent surge in 2024 and 2025. This longitudinal perspective ensures that the synthesis remains contemporary and reflects the specialized evolution of cyberbullying in the post-pandemic era.

### 4.5. Strengths of the Study

A primary strength of this systematic review is the integration of a comprehensive 2026 update, which incorporates 15 high-impact studies into the original 2024 sample, bringing the final corpus to 42 articles. This update provides a critical longitudinal perspective that allows the analysis to transcend the initial post-pandemic transition period observed in 2022. While earlier evidence suggested transient fluctuations in harassment dynamics during that year, the pronounced surge in scientific production during 2024 and 2025 confirms a persistent intensification of the phenomenon and its psychiatric repercussions. Furthermore, the shift in recent publications toward specialized psychiatric and nursing journals underscores the increasing clinical urgency and the necessity of nursing-led interventions to mitigate long-term comorbidities. By synthesizing these recent findings, this review provides the most contemporary and representative global overview of cyberbullying in the current digital era.

## 5. Conclusions

The evidence synthesized in this systematic review, updated to 2026, confirms that cyberbullying is a dynamic and evolving public health crisis with profound psychiatric implications for the adolescent population. Based on the findings, the following conclusions are established:

- Cyberbullying prevalence exhibits significant global variability, with a identified median of 19.1%. This fluctuation is driven by methodological heterogeneity and the emergence of specialized harassment strategies in the post-pandemic era.
- There is a direct and severe relationship between digital victimization and mental health deterioration, characterized by high rates of depression (90%) and anxiety (87%). Most critically, the report of suicide attempts in 19.0% of victims identifies cyberbullying as a primary clinical indicator for psychiatric risk.
- Beyond gender and sexual orientation (LGBTQ+), neurodivergent adolescents—specifically those with autism spectrum disorder—represent a critical risk group, with prevalence rates reaching 68.9%. Interventions must be urgently tailored to address the unique social–digital challenges faced by these populations.
- Bystanders are not passive observers but secondary victims who experience significant psychological distress, including fear and moral frustration. Transforming “passive reinforcers” into “active upstanders” is essential to breaking the cycle of violence.
- Traditional educational models are insufficient for the current digital landscape. The implementation of modernized cyber-education, utilizing interactive tools and “serious games,” is fundamental to fostering digital citizenship and empathy.
- As health leaders, nursing professionals must play a central role in school-based prevention and clinical intervention. A holistic care model focused on emotional regulation and the mitigation of “thwarted belongingness” is necessary to provide effective support for affected youth.

## Figures and Tables

**Table 1:** MeSH terms used in the search.

MeSH Term	Descriptor ID
Adolescent	D000293
Child	D002648
Childhood	Free term
Cyberbullying	D000077224
Mental Health	D008603
Depression	D003863
Anxiety	D001007
Suicide	D013405
Neurodiversity	Free term
Gender Identity	D005783

**Table 2:** Search equations.

Database	Search Date	Search Equation/String	Results (*n*)
PubMed	22 August 2024	(Cyberbullying [Title] OR “Digital bullying” [Title]) AND (“Emotional impact” OR “Psychological effects” OR “Psychological Stress”) AND (“Childhood” OR “Child” OR “Adolescent”)	8
WoS	22 August 2024	TI = (“Cyberbullying” OR “Digital bullying”) AND TI = (“Emotional impact” OR “Psychological effects” OR “Psychological Stress”) AND TI = (“Childhood” OR “Child” OR “Adolescent”)	17
Scopus	22 August 2024	TITLE((“Cyberbullying” OR “Digital bullying”) AND (“Emotional impact” OR “Psychological effects”) AND (“Childhood” OR “Child” OR “Adolescent”))	28
EBSCO	22 August 2024	(“Cyberbullying” OR “Digital bullying”) AND (“Emotional impact” OR “Psychological effects” OR “Psychological Stress”) AND (“Childhood” OR “Child” OR “Adolescent”)	12
PubMed	15 January 2025	(Cyberbullying [Title/Abstract] OR “Online Harassment” OR “Internet Bullying”) AND (Adolescent OR Child OR Youth) AND (“Mental Health” OR Emotions)	182
WoS	15 January 2025	TS = (“Cyberbullying” OR “Online Harassment”) AND TS = (“Adolescent” OR “Children” OR “Youth”) AND TS = (“Mental Health” OR “Emotions”)	16
Scopus	15 January 2025	TITLE-ABS-KEY((“Cyberbullying” OR “Online Harassment”) AND (“Adolescent” OR “Youth”) AND (“Mental Health” OR “Emotions”))	49
EBSCO	15 January 2025	(“Cyberbullying” OR “Online Harassment”) AND (“Adolescent” OR “Children”) AND (“Mental Health” OR “Emotions”)	6
PubMed	20 February 2026	(Cyberbullying [MeSH] OR “online harassment” OR “digital bullying”) AND (“Mental Health” [MeSH] OR “Suicide” [MeSH] OR “Depression” OR “Anxiety”) AND (“Adolescent” [MeSH] OR “Child”)	232
WoS	20 February 2026	TS = (“Cyberbullying” OR “Online Harassment”) AND TS = (“Mental Health” OR “Suicide” OR “Depression” OR “Anxiety”) AND TS = (“Adolescent*” OR “Child*”)	989
Scopus	20 February 2026	TITLE-ABS-KEY((“Cyberbullying” OR “Online Harassment”) AND (“Mental Health” OR “Suicide” OR “Depression” OR “Anxiety”) AND (“Adolescent” OR “Child”))	86
EBSCO	20 February 2026	(“Cyberbullying” OR “Digital bullying”) AND (“Mental Health” OR “Suicide” OR “Depression” OR “Anxiety”) AND (“Adolescent” OR “Child”)	148
TOTAL		Total Records Identified (Gross)	1784

**Table 3:** Prevalence of victims and aggressors by country, perpetrator, And type of harassment.

Country	Author(s)	Type of Harassment	Sample Size (*n*)	Gender Percentage	Victims (%)	Aggressors (%)
Argentina	Dadras, 2024 [46]	Both	56,783	48.0% Male, 52.0% Female	CB: 17.1–48.5%; B: 21.2–46.9%	N.S.
Canada	Kim, 2018 [47]	Both	4940	43.3% Male, 56.7% Female	CB: 19.8%	N.S.
China	Hu, 2025 [66]	Both	582	49.1% Male, 50.9% Female	N.S.	N.S.
	Jiang, 2022 [67]	CB	Not specified	Not specified	N.S.	N.S.
	Liu, 2025 [48]	CB	120	22.0% Male, 78.0% Female	50.0%	N.S.
	Meng, 2023 [68]	Both	497	46.1% Male, 53.9% Female	N.S.	N.S.
	Song, 2024 [49]	CB	344	56.1% Male, 43.9% Female	CB: 31.1%	N.S.
	Wang, 2022 [69]	CB	607	48.9% Male, 51.1% Female	N.S.	N.S.
	Wu, 2025 [70]	CB	460	47.0% Male, 53.0% Female	N.S.	N.S.
China and US	Wright, 2024 [71]	Both	908	CN: 49.0% F, US: 52.0% F	N.S.	N.S.
Colombia	Marín-Cortés, 2020 [65]	CB	31	45.2% Male, 54.8% Female	N.S.	N.S.
Germany	Baier, 2019 [72]	Both	10,502	50.2% Male, 49.8% Female	N.S.	N.S.
Hungary	Arató, 2021 [50]	CB	1105	49.9% Male, 50.1% Female	CB: 12.0%	CB: 6.5%
Italy	Gianesini, 2015 [42]	CB	494	50.8% Male, 49.2% Female	CB: 67.7%	CB: 55.6%
Nepal	Khadka, 2024 [45]	CB	501	100% Female	N.S.	N.S.
Norway	Kaiser, 2020 [51]	Both	2117	50.4% Male, 49.6% Female	CB: 11.8%; B: 12.3%	CB: 4.2%
	Skilbred-Fjeld, 2020 [52]	Both	4531	40.8% Male, 59.2% Female	CB: 4.0%	CB: 2.0%
	Stea 2024 [41]	Both	16,482	49.2% Male, 50.8% Female	CB: ~2.1%; B: ~4.3%	B: ~2.0%
Portugal	Caetano, 2016 [53]	CB	3525	47.8% Male, 52.1% Female	CB: 7.6%	CB: 3.9%
	Gomes, 2024 [73]	CB	140	49.3% Male, 50.7% Female	N.S.	N.S.
Saudi Arabia	Gohal, 2023 [54]	CB	355	32.0% Male, 68.0% Female	CB: 88.0%	CB: 11.0%
Spain	Buelga, 2024 [74]	Both	1007	51.9% Male, 48.1% Female	N.S.	N.S.
	Cabrera, 2024 [55]	Both	1139	Not specified	Rural: 24.8%; Urban: 29.1%	Rural: 10.7%; Urban: 13.4%
	Garaigordobil, 2025 [75]	Both	1748	47.4% Male, 52.6% Female	CB: 7.2%; B: 11.0%	B: 2.7%
Spain and COL	Fajardo-Bullón, 2021 [56]	CB	1510	ES: 48.9% M, 51.1% F; CO: 40.5% M, 59.5% F	ES: 13.1%; CO: 11.7%	ES: 6.1%; CO: 9.6%
Switzerland	Schulz, 2025 [76]	CB	2052	51.0% Male, 49.0% Female	N.S.	N.S.
Taiwan	Chang, 2013 [57]	Both	2992	52.0% Male, 48.0% Female	CB: 29.6%; B: 13.3%	CB: 17.0%; B: 15.7%
	Liu, 2021 [21]	Both	506	16.2% Male, 83.8% Female	CB: 13–18%; B: 20–26%	CB: 8–14%; B: 13–18%
Turkey	Uysal, 2025 [77]	CB	1985	46.8% Male, 53.2% Female	N.S.	N.S.
UK	Fahy, 2016 [58]	CB	2480	55.2% Male, 44.8% Female	CB: 34.0%	CB: 28.6%
US	Accardo, 2025 [43]	Both	71,973	Not specified overall	64.1–68.9% (Autistic youth)	N.S.
	Alhajji, 2019 [59]	CB	15,465	51.3% Male, 48.7% Female	CB: 15.5%	N.S.
	Nagata, 2022 [60]	CB	9429	51.4% Male, 48.6% Female	CB: 9.6%	CB: 1.1%
	Nagata, 2025 [61]	CB	9095	51.3% Male, 48.7% Female	CB: 9.2%	N.S.
	Nicolai, 2018 [62]	Both	137	30.0% Male, 70.0% Female	CB: 41.6%	N.S.
	Duarte, 2018 [44]	CB	1031	47.8% Male, 52.2% Female	CB: 19.1%	CB: 13.6%
	Smokowski, 2014 [63]	Both	3127	47.8% Male, 52.2% Female	CB: 15.0%; B: 31.0%	N.S.

Acronyms: N.S. Not specified; B: Bullying; CB: Cyberbullying.

**Table 4:** Ranking of emotional implications in victims and aggressors.

Emotional/Clinical Implication	Percentage in Victims	Percentage in Aggressors	Reference Study
No reaction/Indifference	30.4%	45.6%	Gianesini, 2015 [42]
Shame/Embarrassment	13.5%	8.9%	
Anger/Rage	39.5–39.7%	N/S	Gianesini, 2015 [42] Caetano, 2016 [53]
Sadness	17.2–44.9%	N/S	
Humiliation	23.6–25.9%	N/S	
Fear	9.5–24.6%	N/S	
Insecurity/Helplessness	16.4–19.9%	N/S	
Guilt/Regret	N/S	10.6–26.6%	
Feeling good/Satisfaction	N/S	15.2–32.6%	
Confidence/Relief	N/S	8.9–26.5%	
Desire for revenge	35.8%	N/S	Caetano, 2016 [53]
Depression *(Clinical score)*	69.6%	50.0%	Skilbred-Fjeld, 2020 [52]
Anxiety *(Clinical score)*	56.6%	56.9%	
Self-harm	28.9%	15.2%	
Suicide attempts	19.0%	3.0%	

Acronyms: N/S, Mentioned as common.

**Table 5:** Differences between the sexes in dealing with traditional bullying and cyberbullying.

Sex	Probability (Prevalence)	Emotional Impact	Types of Harassment	Behavioral Consequences	Anonymity Role	Suicidal Ideation
Female	Tend to report a higher probability of being cybervictims, especially during early and middle adolescence [54,60].	Greater negative impact and internalization; experience higher levels of anxiety, depression, alienation, anger, humiliation, and psychosomatic symptoms [42,47,72,73].	Suffer and perpetrate indirect and relational harassment (spreading rumors and social exclusion). Online, they are targets of sexual harassment, attacks on their appearance, or private photos on social networks (“drama”) [42,72].	Tend to internalize experiences (developing depression or anxiety) and show greater clinical problems and, in some cases, increased substance use [72].	Virtual anonymity fits with female socialization practices, facilitating indirect forms of aggression (rumors without physical confrontation) [52]. As aggressors, they sometimes report relief or confusion [53].	Show significantly higher rates of suicidal ideation and are much more vulnerable to interpersonal stress that triggers these thoughts after cyberbullying [47,59].
Male	Higher probability of being cyberaggressors and physical bullies [60]. Although in some studies or specific countries, they also report high rates in the dual role of bully–victim [57].	Show less overall emotional reactivity and report less initial affectation (indifference) [42]. However, when victimized, the emotions they experience most are a desire for revenge, fear, and sadness [53,78].	Suffer and perpetrate more direct and aggressive harassment [72]. In the cyber environment, attacks usually occur through online video games or through direct insults and homophobic comments (“trash talk”) [42].	Tend to externalize stress, blaming others and becoming aggressive or developing conduct problems [72]. Their victimization has been associated with physical fights and carrying weapons [59].	Anonymity allows them to perpetrate aggression under “online disinhibition” without using physical force [52,58]. In addition, due to gender roles (masculinity), men avoid asking for help and hide that they are victims [59].	Although they report less initial “ideation” than women, in men, cyberbullying strongly predicts suicide planning, and completed suicide rates are much higher [59].

## Data Availability

No new data were created or analyzed in this study. Data sharing is not applicable to this article.

## Supplementary Materials

The following supporting information can be downloaded at: https://www.mdpi.com/article/10.3390/children13030367/s1, Synthesis of results of the articles included in the systematic review, PRISMA 2020 Checklist, Quality Assessment and PRISMA Flow diagram.

## Abbreviations

The following abbreviations are used in this manuscript:

BDI-II	Beck Depression Inventory-II
BSI	Brief Symptom Inventory
CES-D	Center for Epidemiologic Studies Depression Scale
CYBAGRESS	Cyber-Aggression Scale for Mobile Phone and Internet
CYBVYC	Cyber-Victimization Scale for Mobile Phone and Internet
DASS	Depression, Anxiety and Stress Scale
DERS-SF	Difficulties in Emotion Regulation Scale—Short Form
GSHS	Global School-based Student Health Survey
HBSC	Health Behaviour in School-aged Children
HSCL-25	Hopkins Symptom Checklist-25
ICIB	School Cyberbullying Instrument
ICT	Information and Communication Technologies
JBI	Joanna Briggs Institute
KPDS-10	Kessler Psychological Distress Scale
LGBT	Lesbian, Gay, Bisexual, and Transgender
MeSH	Medical Subject Headings
MHI-5	Mental Health Inventory-5
NSSI	Non-Suicidal Self-Injury
OECD	Organisation for Economic Co-operation and Development
OSF	Open Science Framework
PEO	Population, Intervention, Outcome
PRISMA	Preferred Reporting Items for Systematic Reviews and Meta-Analyses
PTSD	Post-Traumatic Stress Disorder
SAS-A	Social Anxiety Scale for Adolescents
SCL-90-R	Symptom Checklist-90-Revised
SDQ	Strengths and Difficulties Questionnaire
SES	Socioeconomic Status
