# Supplementary material for: Study of the Relationship Between Cyberbullying and Mental Health in Adolescents—A Systematic Review
Source: Children (Basel). 2026 Mar 4;13(3):367. doi: 10.3390/children13030367 (PMC13025276; doi:10.3390/children13030367)
Supplement: Supplementary file 1 [file children-13-00367-s001.zip › Quality Assessment.pdf]

Study (Abbreviated Citation)	Study Design	JBI Tool Used	Score	Quality Level
Accardo et al. (2025)	Analytical Cross-Sectional	Checklist for Analytical Cross-Sectional	8 / 8	High
Arató et al. (2021)	Analytical Cross-Sectional	Checklist for Analytical Cross-Sectional	8 / 8	High
Baier (2018)	Cross-Sectional	Analytical Cross-Sectional	8/8	High
Buelga et al. (2024)	Analytical Cross-Sectional	Checklist for Analytical Cross-Sectional	8 / 8	High
Cabrera (2022)	Cross-Sectional	Analytical Cross-Sectional	8/8	High
Caetano (2016)	Cross-Sectional	Analytical Cross-Sectional	7/8	High
Chang (2013)	Cross-Sectional	Analytical Cross-Sectional	7/8	High
Dardas (2014)	Cross-Sectional	Analytical Cross-Sectional	8/8	High
Duarte (2018)	Cross-Sectional	Analytical Cross-Sectional	8/8	High
Fahy (2016)	Cohort	Cohort Studies	9/11	High
Fajardo-Bullón (2021)	Cross-Sectional	Analytical Cross-Sectional	8/8	High
Garaigordobil (2020)	Quasi-experimental	Quasi-Experimental Studies	9/9	High
Garaigordobil (2025)	Prospective Review	Checklist for Text and Opinion	6 / 6	High
Gianesini (2015)	Cross-Sectional	Analytical Cross-Sectional	7/8	High
Gohal (2023)	Cross-Sectional	Analytical Cross-Sectional	7/8	High
Gomes (2024)	Cross-Sectional	Analytical Cross-Sectional	8/8	High
Hu et al. (2025)	Analytical Cross-Sectional	Checklist for Analytical Cross-Sectional	8 / 8	High
Jiang (2020)	Cross-Sectional	Analytical Cross-Sectional	7/8	High
Jiao (2025)	Cross-Sectional	Analytical Cross-Sectional	7/8	High
Kaiser (2020)	Cross-Sectional	Analytical Cross-Sectional	8/8	High
Khadka/ Santosh (2024)	Cross-Sectional Survey	Analytical Cross-Sectional	7/8	High
Kim (2018)	Cross-Sectional	Analytical Cross-Sectional	7/8	High
Liu et al. (2021)	Analytical Cross-Sectional	Checklist for Analytical Cross-Sectional	8 / 8	High
Liu et al. (2025)	Quantitative (Big Data)	Checklist for Analytical Cross-Sectional*	7 / 8	High
Marín-Cortés (2020)	Qualitative	Qualitative Research	10/10	High
Marín-Cortés (2021)	Cross-Sectional	Analytical Cross-Sectional	8/8	High
Meng et al. (2023)	Short-term Prospective	Checklist for Cohort Studies	10 / 11	High
Nagata et al. (2022)	Prospective Cohort	Checklist for Cohort Studies	11 / 11	High
Nagata et al. (2025)	Prospective Cohort	Checklist for Cohort Studies	11 / 11	High
Nicolai (2018)	Cross-Sectional	Analytical Cross-Sectional	8/8	High
Schulz et al. (2025)	Longitudinal Study	Checklist for Cohort Studies	10/ 11	High
Skilbred-Fjeld (2020)	Cross-Sectional	Analytical Cross-Sectional	8/8	High
Smokowski (2014)	Cohort	Cohort Studies	10/11	High
Song et al. (2024)	Analytical Cross-Sectional	Checklist for Analytical Cross-Sectional	8 / 8	High

Stea (2024)	Cross-Sectional	Analytical Cross-Sectional	8/8	High
Tian et al. (2018)	Longitudinal	Cohort Studies	10/11	High
Uysal et al. (2025)	Analytical Cross-Sectional	Checklist for Analytical Cross-Sectional	8 / 8	High
Wang (2021)	Cross-Sectional	Analytical Cross-Sectional	8/8	High
Wright (2024)	Cross-Sectional	Analytical Cross-Sectional	8/8	High
Wu et al. (2025)	Longitudinal Study	Checklist for Cohort Studies	10 / 11	High
Yosep et al. (2025)	Qualitative Study	Checklist for Qualitative Research	10 / 10	High
