# Supplementary material for: Study of the Relationship Between Cyberbullying and Mental Health in Adolescents—A Systematic Review
Source: Children (Basel). 2026 Mar 4;13(3):367. doi: 10.3390/children13030367 (PMC13025276; doi:10.3390/children13030367)
Supplement: Supplementary file 1 [file children-13-00367-s001.zip › Results.pdf]

ABBREVIATED CITATION	STUDY	RESEARCH QUESTION	METHOD	RESULTS	CONCLUSIONS
Tian et al 2018	Design: Observational and longitudinal study Objectives: To examine the mediating role of perceived stress in peer relationships in the association between cyberbullying, cybervictimization, and mental health in early adolescents. Location and period of realization: Study in 4 high schools in northern China over a 3-year period.	Population: 606 adolescents Intervention: No information Comparison: No information Analyzed outcomes: The study focuses on the relationship between cyberbullying, cybervictimization, and mental health in early adolescents, and examines the mediating role of perceived stress in peer relationships in this association. Follow-up time: The longitudinal study was conducted over three years, with students completing questionnaires every 12 months.	No. participants/group: 4 classes within each of the 4 participating schools, but it is not specified whether these classes were used as separate groups for the analysis. Experimental group intervention: No intervention is described. The study is observational and aims to examine the relationship between cyberbullying, cybervictimization, perceived stress in peer relationships, and mental health in adolescents. Control group intervention: No information Blinding method: No information Post-randomization losses: Of 630 initial students, the final population was reduced to 606 due to missing data in the surveys.	Beneficial clinical effects: Cybervictimization has a stronger impact on adolescent mental health than cyberbullying. Perceived stress in peer relationships does not appear to be an important link between mental health and cyberbullying. Adverse effects: No	Cybervictimization has a stronger impact on adolescent mental health than cyberbullying. Perceived stress in peer relationships acts as a mediator between cybervictimization and mental health, but not between cyberbullying and mental health.

ABBREVIATED CITATION	STUDY	RESEARCH QUESTION	METHOD	RESULTS	CONCLUSIONS
Wright, 2024	Design:Prospective cohort study Objectives:To investigate differences in perceived social support from parents and friends between Chinese and American adolescents. To examine whether perceived social support from parents and friends moderates the relationship between cyberbullying victimization and mental health problems. Location and period:Beijing (China) and Chicago (Illinois, USA). Over one school year (fall of seventh grade to fall of eighth grade).	Population:908. Exposure:Cyberbullying victimization, measured using a frequency scale. Clinical effects:The following were assessed: Depression using the CES-D scale. Subjective health complaints using the Youth Self-Report symptom checklist. Self-harm using the self-harm questionnaire.	Number of subjects / group:463 students from Beijing. 445 students from Chicago. Characteristics of exposed cohort:School-enrolled adolescents: The study sample consisted of eighth-grade students from public schools in Beijing (China) and Chicago (Illinois, USA). Age: The age range of participants was 13-15 years. Socioeconomic status: The selected schools were reported to be located in predominantly middle-class neighborhoods. Ethnic composition: American group: 73% White, 20% Latino, 5% African American, 1% Asian, and 1% biracial; for the Chinese group, it is only mentioned that most participants identified with the Han ethnicity. Characteristics of unexposed cohort:No information Exposure factor:Cyberbullying victimization is defined, providing examples of victimization behaviors through the 9-item scale. Type of Comparison:Yes Follow-up period:The study conducts a cross-cultural comparison between two groups of adolescents: one from China and one from the USA, by investigating differences in perceived social support from parents and friends between these two cultural groups and examining how this support moderates the relationship between cyberbullying victimization and depression, subjective health complaints, and self-harm. Losses: n / group:In the initial phase, 10 Chinese participants were	No significant differences were found in perceived parental support between Chinese and American adolescents. American adolescents reported greater perceived friend support compared to Chinese adolescents. In both cultural groups, cyberbullying victimization was positively associated with depression, subjective health complaints, and self-harm over time. Both perceived parental and friend support were negatively associated with depression, subjective health complaints, and self-harm over time. In both cultural groups, perceived parental support moderated the relationship between cyberbullying victimization and depression, subjective health complaints, and	The study confirms the importance of social support, from both parents and friends, as a crucial protective factor against the negative effects of cyberbullying victimization in adolescents. High levels of perceived social support were associated with a reduction in depression, subjective health complaints, and self-harm over time, one year after the initial assessment. The study identified significant differences in how these types of support moderated the relationship between cyberbullying victimization and negative mental health outcomes. In Chinese adolescents, perceived parental support had a stronger moderating effect. This aligns with Chinese cultural values that emphasize family harmony, filial piety, and the central role of parents in children's well-being. In American adolescents, perceived friend support showed a

			lost due to lack of permission and 28 American participants due to denial of permission or absences. In the follow-up phase, 20 Chinese participants and 25 American participants were lost due to logistical reasons. These losses represent 6.14% for Chinese participants and 10.41% for American participants.	self-harm over time. Perceived friend support also moderated the relationship between cyberbullying victimization and negative outcomes over time; this moderating effect was more pronounced in American adolescents than in Chinese adolescents, while parental support was stronger for Chinese adolescents.	more pronounced moderating effect, reflecting the importance of peer relationships and the search for social validation in American culture.

ABBREVIATED CITATION	STUDY	RESEARCH QUESTION	METHOD	RESULTS	CONCLUSIONS
Marín-Cortés 2020	Design: Classified as a cross-sectional case series. Objectives: To understand the experiences of fear and sadness as factors that compromise the mental health of adolescents involved as	Population: 31 students from public and private schools in Medellín formed a non-probability sample. Participants are between 12 and 17 years old, all from middle socioeconomic sectors, 54.8% of whom are women. Intervention or common characteristic: Open and unstructured interviews were conducted	N° cases: 31 students Case criteria: The study uses the School Cyberbullying Instrument (ICIB), developed by Baquero and Avendaño (2015), to select participants. This instrument is validated for the Colombian school-age adolescent population. Follow-up period: Partially N° and percentage lost: No	Although spectators consider that cyberbullying has serious consequences for victims, they do not intervene in these situations because they, in turn, fear being cyber-victimized. Cyberbullying can generate a set of negative emotions in	The study highlights that spectators of cyberbullying are not passive observers but are emotionally, attitudinally, and behaviorally affected. These adolescents experience fear, sadness, guilt, and helplessness, which negatively impacts their mental and social health. The fear of retaliation is key to explaining their

	cyberbullying spectators. Location and period of realization: Medellín, Colombia. 2020	(Schettini and Cortazzo, 2016). Instant messaging platforms Skype, WhatsApp, and Messenger were used for the interviews. The interview protocol contained two central themes: participation in cyberbullying situations and emotions associated with that experience. Effects: The study seeks to characterize the fear and sadness experienced by adolescent spectators of cyberbullying. These effects are relevant from a clinical and social perspective, as they can have negative consequences for the mental health and well-being of young people. Follow-up period: Partially		spectators, including sadness, pain, frustration, anger, and fear. Sadness is also a central emotion in the spectators' experience; this emotion is related to empathy towards the victims.	inaction, while sadness, generated by empathy towards the victims, motivates them to provide emotional support. The study underscores the importance of including spectators in interventions, developing strategies to address their emotions and foster their active role against cyberbullying. It is also recommended to train parents, teachers, and institutions to identify changes in adolescents and promote responsible use of technology.

ABBREVIATED CITATION	STUDY	RESEARCH QUESTION	METHOD	RESULTS	CONCLUSIONS

Marín-Cortés 2021	Design: Cross-sectional case series study Objectives: To understand the experiences of compassion, jealousy, and envy of adolescents involved in cyberbullying situations in the roles of spectators and aggressors Location and period of realization: It was carried out in several public and private schools in Medellín, Colombia 2017	Population: 31 adolescents Intervention or common characteristic: Screening was carried out with the School Cyberbullying Instrument ICIB: The ICIB (Baquero & Avendaño, 2015) was applied to select participants who had been involved in cyberbullying situations. The main "intervention" of the study was the conduct of unstructured interviews through instant messaging applications (Messenger, WhatsApp, and Skype). Effects: The study focuses on identifying and analyzing the consequences of cyberbullying on the psychological and emotional well-being of spectators. Follow-up period: No	Nº cases: 31 adolescents Case criteria: School-age adolescents, from middle-income sectors, residing in Medellín, Colombia, between 12 and 17 years old, who reported having been involved in cyberbullying situations. Those who did not meet these criteria were excluded Follow-up period: Yes Nº and percentage of losses: No	The results indicate that compassion takes the form of sadness and anger, especially among cyberbullying spectators. On the other hand, jealousy is a motive that justifies aggression towards others through the Internet, and a way to manage tensions in sexual-affective bonds. The use of digital social networks and participation in face-to-face scenarios where positions are disputed at an academic level favors the process of social comparison linked to the envy that is at the basis of cyberbullying.	The study highlights the importance of raising awareness and denaturalizing cyberbullying for everyone involved, including spectators. The need to promote self-care and care for others, solidarity, and cooperation among peers to prevent and stop cyberbullying and promote the mental health of adolescents is emphasized.

ABBREVIATED CITATION	STUDY	RESEARCH QUESTION	METHOD	RESULTS	CONCLUSIONS

Kaiser 2020	Design: Cross-sectional cohort study<br>Objectives: Examine possible sex differences in the relationship between cyberbullying status and outcome variables (mental health). Location and period of realization: Location: Three counties in Northern Norway (Nordland, Troms, and Finnmark). Period: Spring 2017.	Population: The target population is 2,117 secondary school students. Exposure: The study defines the exposure factor as cyberbullying status.<br><br>Clinical effects: The study adequately defines the clinical effects under study, focusing on adolescent mental health. To assess this aspect, researchers used the Strengths and Difficulties Questionnaire.	Number of subjects / group: Not involved: 1,781 adolescents. Cybervictim only: 179 adolescents. Cyberaggressor only: 23 adolescents. Cybervictim and aggressor: 64 adolescents. Characteristics of exposed cohort: The variables studied in the exposed population are: School-enrolled adolescents. Age range. Sex. Nationality. Experience of traditional bullying. Characteristics of unexposed cohort: No information. Exposure factor: Defines the exposure factor, called "cyberbullying status," using criteria based on the frequency of victimization and aggression in cyberspace.<br><br>Type of Comparison: Yes. Follow-up period: It is a cross-sectional study; there is no follow-up. Losses: number / group: The response rate was 40%.	Adolescents who were both cybervictims and cyberbullies, as well as those who were only cybervictims, reported more mental health problems than those not involved in any form of cyberbullying. These two groups scored highest on the total difficulties score, the emotional symptoms scale, the hyperactivity/inattention scale, and the peer problems scale. Girls' mental health appears to be more affected than boys' when exposed to or involved in cyberbullying. Girls with no experience of cyberbullying scored higher on the total difficulties score, the emotional symptoms scale, and the prosocial behavior scale than boys. Girls who had been cybervictims or were both cybervictims and cyberbullies scored even higher on the total difficulties score and the emotional symptoms scale. Girls who were only cybervictims scored higher on the hyperactivity/inattention and peer problems scales compared to boys.	Cyberbullying negatively affects adolescent mental health. Girls are more vulnerable to the effects of cyberbullying.

SHORT CITATION	STUDY	RESEARCH QUESTION	METHOD	RESULTS	CONCLUSIONS
Wang 2022	Design: Cross-sectional cohort study with grade-based cluster random sampling. Objectives: To examine the mechanisms through which cyberbullying victimization affects the mental health of adolescents in China. Location and period of realization: Ningbo, Zhejiang province (China).	Population: The study population is composed of 607 adolescents (297 males and 310 females). Exposure: The exposure factor is cyberbullying victimization studied through the Wong and McBride Cyberbullying Victimization Scale. Clinical Effects: The study defines the clinical effects under study as social anxiety and depression.	Number of subjects / group: A total of 607 adolescents are included; no information appears between the exposed and unexposed groups. Exposed cohort characteristics: The variables used are: Age. Cyberbullying victimization. Social competence. Unexposed cohort characteristics: No information. Exposure factor: No information. Type of Comparison: No information. Follow-up period: Cross-sectional study with a single data collection period. Losses: No. / group: No information.	Positive Relationship between Cyberbullying Victimization and Symptoms of Depression and Social Anxiety. Mediating Effect of Self-Control. Absence of Mediating Effect of Social Competence.	Cyberbullying victimization is a significant risk factor for depression and social anxiety in adolescents. Self-control plays a crucial mediating role in the relationship between cyberbullying victimization and mental health. Social competence, although negatively related to social anxiety, does not have a significant mediating effect on the relationship between cyberbullying victimization and mental health.

SHORT CITATION	STUDY	RESEARCH QUESTION	METHOD	RESULTS	CONCLUSIONS

Ortega 2009	Design: Descriptive cross-sectional cohort study $\newline$ Objectives: Describe the prevalence of different types of victimization. Analyze the emotional impact reported by victims of both types of bullying (traditional and cyberbullying) and establish differential emotional profiles. Location and period of realization: Córdoba (Spain) between 2006 and 2008	Population: The final sample of the study consisted of 1,671 adolescents. 1st year of Compulsory Secondary Education: 539 students (12-13 years old) 3rd year of Compulsory Secondary Education: 534 students (14-15 years old) 1st year of Baccalaureate: 598 students (16-17 years old) Exposure: Victimization by bullying (direct and indirect) both traditional and cyberbullying (via mobile phone or internet). Defines two levels of severity for the exposure factor; occasional or severe victimization. $\newline$ $\newline$ Clinical effects: The study uses the term "emotional impact" to refer to the emotions and feelings experienced by bullying victims although it does not seek to diagnose mental or psychological disorders.	Number of subjects / group: 1671 adolescents. Not exposed: 1496 Exposed: 175 Characteristics of the exposed cohort: Nationality. Age. Educational level. Gender. Type of Bullying. Severity of bullying. Characteristics of the non-exposed cohort: Nationality. Age. Educational level. Gender. Exposure factor: The study explores 4 types of bullying: Direct bullying. Indirect bullying. Mobile cyberbullying. Internet cyberbullying: $\newline$ $\newline$ Type of Comparison: Comparison between Types of Bullying. Comparison according to Bullying Severity. Comparison according to Gender. Comparison according to Age. Follow-up period: No information Losses: No. / group: Out of 1,755 students, but data from 4.9% of the participants were excluded, a total of 86 students	The study found that traditional bullying (direct and indirect) is more common than cyberbullying. Victimization by bullying decreased significantly between 12 and 17 years old. Men were more likely to be victims of direct bullying, while women reported more cases of cyberbullying. Anger was the most common emotional response, especially in direct bullying. Five different emotional profiles were identified in victims of direct bullying, while only two profiles were found in indirect bullying and cyberbullying. Victims suffering from severe bullying reported more feelings of shame, stress, discomfort, depression, and loneliness.	Cyberbullying is similar to traditional indirect bullying in its emotional impact. Anger is a key emotion in the response to bullying. Direct bullying generates a greater variety of emotional responses. Emotional response. The severity of bullying influences the emotional impact, especially in direct bullying. Women tend to report more negative emotions. Age does not seem to significantly influence the emotional response.

SHORT CITATION	STUDY	RESEARCH QUESTION	METHOD	RESULTS	CONCLUSIONS
Gianesini 2015	Design: Cross-sectional cohort study. Objectives: To explore emotion differentiation and regulation in adolescents in reaction to cyberbullying perpetration and victimization. To understand the mediating role of resilience in the relationship between adolescents' pathogenic relational experiences and behavioral outcomes. To propose a comprehensive resilience approach for cyberbullying, including developmental, ecological, relational, and self-regulation aspects. To develop specific interventions for victims and aggressors, based on a better understanding of the	Population: The study focuses on 494 Italian middle and high school students, aged 13 to 19, attending different public schools in the city of Bologna. Exposure: The exposure factor is cyberbullying, with different levels of exposure: Victimization (Frequency and intensity, types of victimization, duration, risk level). Perpetration (Frequency and intensity, types of cyberbullying, duration, risk level). Participation Roles. Clinical effects: The authors focus on evaluating: Emotional Regulation (Emotional differentiation, emotional reactivity, emotional regulation strategies). Socioemotional Adjustment (Emotional	Number of subjects / group: Exposed (330) Pure Victims: 89 students (20.6%)? Pure Cyberbullies: 37 students (8.5%)? Cyberbully-Victim: 204 students (47.1%)? Not exposed (103) Not involved: 103 students (23.3%) Exposed cohort characteristics: Italian middle and high school students exposed to cyberbullying, either as victims or perpetrators, the following variables are studied: Gender. Age. Emotional Regulation. Participation Roles. Socioemotional Adjustment. Non-exposed cohort characteristics: No information Exposure factor: No information Type of Comparison: No information Follow-up period: The research was conducted during the 2013-2014 academic year Losses: n / group: No information	Gender Differences: Boys showed higher levels of overall resilience (RS-14) and in the alienation subscale. Girls scored higher on the positivity subscale of dispositional resilience. Girls experienced more anger and humiliation than boys as victims of cyberbullying. Girls were more likely to have been victims of cyberbullying in previous years. Girls perceived a greater impact of their cyberbullying actions on victims than boys. Impact of Cyberbullying: Victimization rates (63.7%) were higher than perpetration rates (51.7%). Victim-perpetrator cyberbullying was prevalent (47.1%). Victims primarily experienced passive emotions (sadness, humiliation, shame), while perpetrators experienced negative emotions (guilt and shame). A moderate level of resilience was sufficient to protect adolescents from engaging in cyberbullying. Resilience and Adjustment: Students with positive adjustment (prosocial behavior) tended to have higher levels of resilience and positivity, and low levels of peer violence, both in perpetration and victimization.	The study underlines the importance of resilience and emotional regulation in coping with cyberbullying in adolescents. It emphasizes the need to consider gender differences and developmental stage when designing interventions and prevention programs.

	emotional impact of peer cyberviolence and resilience after victimization and perpetration. Location and period: Bologna (Italy) between 2013-2014	symptoms, prosocial behavior, psychological well-being).		Students who were maladjusted and at risk due to emotional symptoms had higher scores on alienation and victimization, and low scores on resilience and positivity. Cluster Analysis: Five distinct patterns of resilience and cyberbullying involvement among adolescents were identified: Resilient Victims (RV): High resilience, average positivity, and low alienation. Healthy Uninvolved (HU): Average resilience, average positivity, and extremely low alienation. Healthy Bullies (HB): Average resilience, average positivity, and low alienation. Alienated Victim-Bullies (ABV): Low positivity, very low resilience, and extremely high alienation. Resilient Victim-Bullies (RBV): High positivity, extremely high resilience, and average alienation.	

SHORT CITATION	STUDY	RESEARCH QUESTION	METHOD	RESULTS	CONCLUSIONS

Giménez 2015	Design: Cross-sectional cohort study. Objectives: To examine whether cyberbullies' beliefs about the impact of their actions accurately reflect the impact reported by the cybervictims themselves. To test whether emotional reactions to cyberbullying differ depending on whether the victim has also been a victim of traditional bullying. Location and period of completion: Murcia (Spain). Between 2012 and 2014.	Population: A population of 1353 students between 12 and 20 years old, with 47.3% males. Of these, 1170 (86.5%) were in compulsory secondary education and 183 (13.5%) in non-compulsory education. Exposure: Cyberbullying is defined as the exposure factor, considering the following variables: Frequency. Medium used. Role. Clinical Effects: The study uses an emotion-based approach. To evaluate the emotional impact of cyberbullying, researchers developed a scale that measures five negative emotions: Offended. Helpless. Rejected. Sad. Scared.	Number of subjects/group: Exposed group: 104 students reported being involved in cyberbullying. Of these, 68 were cybervictims, 20 were cyberbullies, and 16 were both. Non-exposed group: 1249 students. Exposed cohort characteristics: Roles in cyberbullying (Cybervictims, 68 students, cyberbullies, cyberbully-victim) Age. Gender. Experience of traditional bullying. Emotions. Non-exposed cohort characteristics: No information. Exposure Factor: Student self-report via the CYBERBULL questionnaire is used to determine participation in cyberbullying. Before asking about their participation, students were provided with a definition of cyberbullying. The questionnaire inquires about cyberbullying via mobile phones and computers. Students are asked about the frequency of cyberbullying incidents in the last month and, in the case of victims, about the duration of victimization. Type of Comparison: Comparison exists between: The perception of emotional impact between cyberbullies and cybervictims. Emotional reactions to	Cyberbullies tend to believe their actions have a greater negative emotional impact on victims than the victims themselves report. Students who reported being victims of both traditional bullying and cyberbullying perceive a greater negative emotional impact from cyberbullying compared to those who only experienced cyberbullying. With the exception of the emotion of "sadness," where girls scored higher than boys, the study found no significant gender differences in the perception of the impact of cyberbullying. A decrease in the frequency of cyberbullying was observed as students' age increases, which coincides with other studies.	Cyberbullies tend to overestimate the negative impact of their actions. Victims of cyberbullying who also suffer traditional bullying experience a greater negative emotional impact. The emotional impact of cyberbullying does not show significant gender differences. Except for the emotion of "sadness," where girls reported higher levels than boys. The frequency of cyberbullying decreases with age.

			cyberbullying in victims of traditional bullying and cyberbullying vs. victims of cyberbullying only. Perceptions of the impact of cyberbullying based on role (cyberbully, cybervictim, cyberbully/victim). Follow-up period: It is a cross-sectional assessment. Losses: number/group: No information.		

SHORT CITATION	STUDY	RESEARCH QUESTION	METHOD	RESULTS	CONCLUSIONS
Nicolai 2018	Design: Cross-sectional design using a survey. Objectives: Main objective: To investigate symptoms of depression, anxiety, and stress in cyberbullying victims who stutter. Specific objectives: To identify if there is a significant difference in depression, anxiety, and stress levels among four defined groups. To	Population: Young adults, aged 18 to 30, who experienced cyberbullying during adolescence. Exposure: Cyberbullying experienced during middle and/or high school, generating levels based on the exposure factor: Cyberbullying present. Cyberbullying absent. Clinical effects: Focuses on investigating	Number of subjects / group: Cyberbullying exposed groups: A total of 57 participants (24 in CB + ST and 33 in CB + No ST) were classified as exposed to cyberbullying. Non-cyberbullying exposed groups: A total of 80 participants (38 in No CB + ST and 42 in No CB + No ST) formed the control groups, with no history of cyberbullying. Exposed cohort characteristics: Age: Between 18 and 30 years old. Participants who stutter must have been diagnosed by a professional (pediatrician, general practitioner, speech therapist, etc.). The cyberbullying must have occurred during this period and for a longer time than traditional bullying (if any). For participants who stutter, most of the cyberbullying must have been directed at their speech, not at other disabilities or characteristics. Absence of other traumas: Non-exposed cohort	The study confirms that cyberbullying and stuttering, individually and combined, can have a negative impact on the mental health of young adults. Anxiety stands out as a particularly affected factor in people who have experienced cyberbullying, especially in those who also stutter. The results underscore the importance of early intervention and social support to mitigate the	There is a significant relationship between cyberbullying, stuttering, and mental health. Cyberbullying can have lasting effects on mental health, even in the absence of stuttering. Stuttering can also be associated with higher stress levels, even without cyberbullying. It is essential to consider the interaction between cyberbullying and stuttering when addressing mental health.

	compare the levels of depression, anxiety, and stress in the group that stutters and has been a victim of cyberbullying with the other three groups. To compare the levels of depression, anxiety, and stress in the control group (no stuttering or cyberbullying) with the other three groups. Location and period of completion: Online data collection study.	symptoms of depression, anxiety, and stress in young adults who experienced cyberbullying during adolescence and who stutter, using the Depression, Anxiety, and Stress Scale.	characteristics Partially Exposure factor: Cyberbullying must have occurred during adolescence, specifically during middle and high school. If the participant also experienced traditional bullying, cyberbullying must have lasted longer. Types of cyberbullying: Text messages, social networks, emails, etc. Frequency of cyberbullying: How often cyberbullying occurred. Perception of cyberbullying: How the participant perceived the experience (threatening, humiliating, etc.). Target of cyberbullying: Most of the cyberbullying must have been directed at the participant's speech, not at other disabilities or characteristics. Type of Comparison: Independent Variables: Cyberbullying. Stuttering. Dependent Variable: Scores on the DASS scale. Comparison Groups: Participants who experienced cyberbullying and stutter. Participants who experienced cyberbullying but do not stutter. Participants who did not experience cyberbullying but stutter. Participants who did not experience cyberbullying or stutter (control group). Statistical Analysis: A two-way between-groups MANOVA was used to determine if there are significant differences in depression, anxiety, and stress scores among the four groups. Post-hoc comparisons were performed to analyze specific differences between each pair of groups. Follow-up period: No information Losses: No. / group: 358 people started the online survey. Incomplete Surveys: 4. Inclusion Criteria: 89 participants were excluded for not meeting the inclusion criteria. Final Number of Participants: 137 participants completed the survey Loss Calculation: Total exclusions: 93 participants (26%).	negative effects of cyberbullying and stuttering on mental health.	

SHORT CITATION	STUDY	RESEARCH QUESTION	METHOD	RESULTS	CONCLUSIONS
Cabrera 2022	Design: Observational analytical cross-sectional study. Objectives: Main objective: To analyze the differences in bullying behaviors (traditional bullying and cyberbullying) between students from secondary schools located in rural and urban areas. Specific objectives: To analyze bullying behaviors (traditional bullying and cyberbullying) in adolescents from urban and rural areas, and to explore the impact of context, gender, and age on bullying. To study the differences in adolescent well-being based on	Population: 1094 secondary school students (ESO and Bachillerato) from 6 secondary schools in Castilla-La Mancha (Spain). Of the total sample, 62.5% of students come from urban settings and 37.6% from rural settings. Exposure: The main exposure factor is the context in which the school is located (rural vs. urban). Urban context: Localities with at least 10,000 inhabitants, a high population density (more than 150 inhabitants/km2), and a high level of infrastructure. Rural context: Localities that do not meet the conditions mentioned above. The study compares the differences in the frequency and intensity of bullying (traditional bullying and cyberbullying) between secondary school students located in these two contexts. In addition to	Number of subjects / group: Exposed: Students from schools located in urban contexts. Not exposed: Students from schools located in rural contexts. The total sample size was 1094 students. Of these: 683 students (62.5%) came from urban settings (exposed). 411 students (37.6%) came from rural settings (not exposed). Characteristics of the exposed cohort: The exposed individuals are secondary school students (ESO and Bachillerato) who attend schools located in urban contexts. The study defines "urban context" using the definition of the National Institute of Statistics of Spain, which characterizes it by: At least 10,000 inhabitants in the locality. High population density (more than 150 inhabitants/km2). High level of infrastructure. Characteristics of the non-exposed cohort: The non-exposed individuals in the study are secondary school students (ESO and Bachillerato) who attend schools located in rural contexts. The rural context is characterized by: Less than 10,000 inhabitants in the locality. Low population density (less than 150 inhabitants/km2). Lower level of infrastructure compared to urban areas. Exposure factor: empty Type of Comparison: The study compares two	A positive and statistically significant correlation was found between cyberbullying and traditional bullying, both in victimization and perpetration. The lowest correlation was observed between physical bullying and cyberbullying. No significant differences were found between rural and urban schools in terms of the total frequency of bullying victimization or perpetration. Nor were significant differences found in the frequencies of participation roles in bullying (victim, aggressor, and victim/aggressor) or in polybullying. Significant differences were found in physical perpetration, being higher in urban schools. Greater victimization by physical bullying was also observed in urban	The size of the population where the schools are located may be a relevant factor for intervention. Intervention strategies should address the general culture of the school and the community, integrating parents and teachers. It is crucial to provide the necessary resources for intervention in rural settings. Cyberbullying is not a separate phenomenon from traditional bullying.

	bullying roles and the context in which the school is located. Location and period of realization: Study conducted in 6 secondary schools in the region of Castilla-La Mancha (Spain).	context, other factors considered include: Gender. Age. Clinical effects: The main clinical effect under study: psychological distress, using the Kessler Psychological Distress Scale K10 (KPDS-10). It considers the influence of the student's role in bullying (victim, aggressor, victim/aggressor).	groups of secondary school students: Exposed group: Students attending schools located in urban contexts. Non-exposed group: Students attending schools located in rural contexts. The comparison is made in terms of different outcome variables related to bullying: Frequency and intensity of victimization and perpetration in different forms of bullying (physical, verbal, social bullying, and cyberbullying). Participation in bullying roles: Victim, aggressor, and victim/aggressor. Level of psychological distress. The study uses different statistical methods for the comparison: Pearson's correlation, Student's t-test, Chi-square test, Multinomial logistic regression, and Analysis of Variance (ANOVA). Follow-up period: No information Losses: n / group: No information	schools. Regarding the targets of bullying, students in rural schools target their classmates more than students in urban schools. Male gender was associated with greater participation in the roles of aggressor and victim/aggressor. Age was negatively associated with victimization, meaning younger age, higher probability of being a victim. The urban context was associated with a higher probability of bullying perpetration. Bullying victims in rural contexts perceive more distress than victims in urban contexts.	

**ABBREVIATED CITATION**	**STUDY**	**RESEARCH QUESTION**	**METHOD**	**RESULTS**	**CONCLUSIONS**	**NOTES**

Gohal 2023	Design: Descriptive cross-sectional study. Objectives: Main objective: Evaluate the prevalence of cyberbullying among adolescents in the Jazan region, Saudi Arabia. Secondary objectives: Identify risk factors associated with cyberbullying. Evaluate the psychological impact of cyberbullying on adolescents. Location and period: Jazan region (Kingdom of Saudi Arabia). Data collection was conducted using a self-administered online questionnaire from May to December 2021.	Population: Specified as adolescents (12-18 years old) who use the Internet to communicate in the Jazan region, Saudi Arabia. The total population used is 355 participants. Exposure: The exposure factor could be the variables that might be associated with cyberbullying: Gender. Age. Educational level. Place of residence. Mother's educational level. Mother's and father's occupation. Time spent on electronic devices. Prior experience as a cyberbullying victim. Frequency of bullying. Affected academic performance. Thoughts of dropping out of school. Thoughts of self-harm. Clinical Effects: The study focuses on the psychological impact of cyberbullying on adolescents. To	Number of subjects / group: Exposed subjects (cyberbullying victims): The total prevalence of cyberbullying was estimated at 42.8%. This means that 152 participants reported having been victims of cyberbullying. Non-exposed subjects: The remaining 57.2% of participants (203) did not report having experienced cyberbullying. Characteristics of the exposed cohort: The exposure factor could be the variables that might be associated with cyberbullying: Gender. Age. Educational level. Place of residence. Mother's educational level. Mother's and father's occupation. Time spent on electronic devices. Prior experience as a cyberbullying victim. Frequency of bullying. Affected academic	The study found an overall prevalence of cyberbullying estimated at 42.8%, with a male prevalence slightly higher than female. 26.3% of participants were significantly affected in their academic performance due to cyberbullying. Approximately 20% of all participants considered leaving their schools, 19.7% considered stopping the use of the Internet, and 21.1% considered self-harm due to the consequences of cyberbullying. Essential links exist between the frequency of bullying, the effect on academic performance, and being a cyber victim. The study confirmed that multiple occurrences of cyberbullying and the possibility of being a	Cyberbullying presents a high prevalence among adolescents in the Jazan region, Saudi Arabia, with significant associated psychological effects. The study highlights the urgency of collaboration between authorities and the community to protect adolescents from this harmful phenomenon. The study emphasizes the significant relationship between cyberbullying and the mental health of adolescents. The study recommends increasing awareness about cyberbullying among parents, educators, and health professionals.	

		assess this impact, the study uses the Mental Health Inventory-5 (MHI-5) questionnaire, which measures two dimensions of mental health: Positive aspects: satisfaction, interest, and enjoyment of life. Negative aspects: anxiety and depression. In addition to the MHI-5, the study also considers other indicators of the impact of cyberbullying, such as: Thoughts of dropping out of school due to cyberbullying. Considering stopping the use of electronic devices. Considering self-harm. Impact on academic performance.	performance. Thoughts of dropping out of school. Thoughts of self-harm. Characteristics of the non-exposed cohort: Yes. Exposure Factor: Yes. Type of Comparison: No information. Follow-up Period: No information. Losses: number / group: The study mentions that surveys were distributed to approximately 385 students. However, the number of respondents who completed the questionnaire was 355, representing a response rate of 92%.	victim are risk factors that influence mental health. Nearly half of the participants wished they could get rid of the perpetrators. More than 20% of the participants considered self-harm due to cyberbullying. Up to 20% of participants considered leaving their schools due to the adverse effects of cyberbullying and wished they could stop using the Internet. 26% of participants felt that their school performance was affected due to being cyber victims.		

Ortega 2012	Design: Cross-sectional cohort study. Objectives: Report the prevalence of victimization for four different types of bullying. Evaluate the emotional impact of the four types of bullying on victims. Identify and characterize the emotional profiles or patterns of victims for each type of bullying. Evaluate the relationship between variables traditionally considered in the field of bullying and emotional profiles. Location and period: The study was conducted in Bologna, Ferrara, and Forlì (Italy), Córdoba (Spain), and London, Midlands (England). Data collection was carried out in late 2007 and early 2008.	Population: Described as adolescents attending secondary schools in three European countries: Italy, Spain, and England. Total size: 5,862 students. Distribution by country: Italy: 1,964 students. Spain: 1,671 students. England: 2,227 students. Mean age: 14.20 years (standard deviation = 1.77). Distribution by school grade: 8th grade (equivalent to 2nd year of ESO in Spain): mean age of 12.24 years. 10th grade (equivalent to 4th year of ESO in Spain): mean age of 14.34 years. 12th grade (equivalent to 2nd year of Baccalaureate in Spain): mean age of 16.38 years. Gender: 48.8% of participants were female. Exposure: The exposure factor is defined as victimization by	Number of subjects / group: Direct Traditional Bullying: Non-exposed (non-victims): 4,999. Exposed (victims): 845. Indirect Traditional Bullying: Non-exposed (non-victims): 4,707. Exposed (victims): 1,114. Cyberbullying via Mobile Phone: Non-exposed (non-victims): 5,438. Exposed (victims): 338. Cyberbullying via the Internet: Non-exposed (non-victims): 5,387. Exposed (victims): 406. Characteristics of the exposed cohort: empty. Characteristics of the non-exposed cohort: empty. Exposure Factor: The exposed individuals in the study are adolescents who reported having been victims of some type of bullying in the last two months. General Characteristics: Age. Gender. Nationality.	In the three countries studied (Italy, Spain, and England), the proportion of traditional bullying victims (direct and indirect) was higher than that of cyberbullying victims (via mobile phone or Internet). Significant differences were found in the prevalence of victimization according to the country. Spain presented the lowest rates of victimization, especially in the case of in-person bullying. England showed a higher prevalence of frequent victimization in general compared to Spain. Italy presented a significantly higher percentage of victims of direct, indirect, and mobile phone bullying compared to Spain. The most reported emotion by students, in both traditional bullying and	Both traditional bullying and cyberbullying are significantly present in the three countries studied: Italy, Spain, and England. The prevalence of traditional bullying, both direct and indirect, was higher than that of cyberbullying in all three countries. Victims of different types of bullying experience distinct emotional responses. Direct bullying and cyberbullying via mobile phone provoke similar emotional profiles, as do indirect bullying and cyberbullying via the Internet. In all types of bullying and in all three countries, the most frequently reported emotion by victims was anger. In all types of bullying, a group of victims was found who reported not being bothered by the situation. Gender,	

		bullying, specifying 4 different types: Direct Traditional Bullying. Indirect Traditional Bullying. Cyberbullying via Mobile Phones. Cyberbullying via the Internet. It also defines two levels of victimization: Occasional Victimization. Frequent Victimization. Clinical Effects: The study focuses on the general emotional impact experienced by victims of bullying and cyberbullying. Instead of evaluating clinical diagnoses such as depression or anxiety, the study focuses on the emotions students report feeling as a consequence of victimization.	Specific Characteristics according to the Type of Bullying: Direct Traditional Bullying. Indirect Traditional Bullying. Cyberbullying via Mobile Phone. Cyberbullying via the Internet. Type of Comparison: The study performs multiple comparisons to analyze the emotional impact of bullying and cyberbullying on adolescents. Comparison between Types of Bullying. Comparison between Countries. Comparison between Demographic Groups. Comparison between Emotional Profiles. To perform these comparisons, the study uses a variety of statistical methods, including: Chi-square tests to analyze the relationship between categorical variables. Z tests to compare	cyberbullying, was anger (with the exception of Spanish cyberbullying victims). The least frequent emotions reported varied according to the country and the type of bullying. In general, the proportion of victims who reported negative emotions in cyberbullying was lower than in traditional bullying. Two main types of emotional profiles were identified: Profile 1: Associated with direct bullying and cyberbullying via mobile phone, with three degrees of harm: unaffected, moderately affected, and strongly affected. Profile 2: Associated with indirect bullying and cyberbullying via the Internet. Girls were more likely to be emotionally affected by indirect bullying and cyberbullying via the Internet. Younger	school grade, country, and frequency of bullying were significantly related to the victims' emotional profile. Girls, younger students, and frequent victims were more likely to experience a greater emotional impact. The cross-sectional design of the study limits the understanding of the directionality of the relationships between the variables. Longitudinal studies are needed to better understand the evolution of emotional impact over time. Further development of measurement instruments is required to assess cyberbullying more accurately and completely. The study highlights the importance of implementing prevention and intervention programs to reduce the incidence of bullying	

			proportions. Hierarchical cluster analysis to identify groups of individuals with similar characteristics. Logistic regression to analyze the influence of independent variables on the probability of belonging to a certain group or profile. Follow-up Period: No information. Losses: number / group: No information.	students were more likely to be affected than older ones. English victims were more likely to be affected than Spanish victims in direct bullying and cyberbullying via mobile phone. Frequent victims were more likely to be affected than occasional victims in all types of bullying.	and cyberbullying. Specific strategies are needed to help victims develop coping skills and resilience.	

Wang 2023	Design: Cross-sectional cohort design. Objectives: The study focuses on the relationship between cyberbullying victimization and non-suicidal self-injury in adolescents. Location and period: The study was conducted in seven primary and secondary schools located in a city in northwestern China. The article does not provide exact dates for data collection.	Population: Described as a group of 2,864 adolescents in grades 5 through 8 in seven primary and secondary schools in a city in northwestern China. Exposure: The exposure factor is cyberbullying victimization. The cyber-victimization subscale is used to assess experiences of cyber-victimization during the 6 months prior to the study. Clinical Effects: Examines two main clinical effects: Social Anxiety: defined as a mental health disorder characterized by an excessive fear of social interactions. Non-Suicidal Self-Injury (NSSI): refers to the deliberate destruction of one's body tissue without lethal intent, a socially unacceptable behavior.	Number of subjects / group: The total study sample is 2,864 adolescents. It is indicated that 17.6% of the participants reported at least one NSSI behavior in the 6 months prior to the study. Characteristics of the exposed cohort: The described characteristics of the exposed individuals are: Age. Sex. Parents' Educational Level. Socioeconomic Level. Origin. Characteristics of the non-exposed cohort: Yes. Exposure Factor: The exposure factor is defined and the cyber-victimization subscale of the second revision of the Revised Cyberbullying Inventory (RCBI-II) is used. Type of Comparison: No information. Follow-up Period: No information. Losses: number / group: No information.	Cyber-victimization is a risk factor for NSSI in adolescents. Social anxiety acts as a mechanism that partially explains this relationship. Emotional reactivity intensifies the relationship between cyber-victimization and NSSI, and between social anxiety and NSSI. Adolescents with high emotional reactivity are particularly vulnerable to the negative effects of cyber-victimization and social anxiety, showing a greater risk of NSSI.	The study confirms that cyber-victimization is an important predictor of NSSI in adolescents. The study provides evidence that social anxiety partially mediates the relationship between cyber-victimization and NSSI. Emotional reactivity plays a crucial role in the relationship between cyber-victimization and NSSI. The study reveals that the relationship between cyber-victimization and NSSI is significantly stronger in adolescents with high emotional reactivity. It is essential to address cyber-victimization through strategies that promote responsible internet use, education on the risks of cyberbullying, and the creation of safe digital environments. The inclusion of emotional regulation	

					training programs in school curricula could be an effective measure to reduce emotional reactivity and prevent NSSI in adolescents.	

Alhaji 2019	Design: Retrospective cross-sectional cohort. Objectives: Examine the association between cyberbullying victimization and mental health problems and violent behaviors in American high school students. Location and period: The location of the study is the United States and the period of realization is 2015.	Population: The study population is composed of 15,465 students in grades 9-12 in public and private schools in the United States. Variables such as: Age. Sex. Race. Ethnicity. Exposure: Defines the exposure factor as cyberbullying victimization. Clinical Effects: Mental health problems: Depressive symptoms. Suicidal ideation. Suicide planning. Violent behaviors: Weapon carrying. Involvement in physical fights. Considerations on the definition of clinical effects: Dichotomous scales. Lack of specificity in violent behaviors.	Number of subjects / group: Total sample: 15,465 students. Non-exposed (non-cyberbullying victims): 13,197 students (84.5% of the sample). Exposed (cyberbullying victims): 2,268 students (15.5% of the sample). Characteristics of the exposed cohort: Demographic Characteristics: Sex. Race. Mental Health Characteristics: Depressive symptoms. Suicidal ideation. Suicide planning. Violent Behaviors: Weapon carrying. Involvement in physical fights. Additional Considerations: Directionality of the association. Characteristics of the non-exposed cohort: Yes. Exposure Factor: The exposure factor is cyberbullying victimization, determined through a	More than 15% of students reported having been victims of cyberbullying at least once in their lives. Women were twice as likely to report victimization as men. Non-white students were 50% less likely to report cyberbullying victimization. Cyberbullying victimization was significantly associated with depressive symptoms, suicidal ideation, suicide planning, weapon carrying, and involvement in physical fights. Women with suicidal ideation were twice as likely to report cyberbullying victimization compared to women without suicidal ideation. Suicide planning and weapon carrying were significantly associated with cyberbullying victimization only	Adolescent girls and white students are at a higher risk of being cyberbullying victims. Cyberbullying victimization is significantly associated with mental health problems and violent behaviors. The negative effects of cyberbullying victimization may be more pronounced in men. Intervention programs against cyberbullying that are sensitive to demographic differences are needed.	

			dichotomous (Yes/No) question about experiencing some form of bullying. Type of Comparison: To achieve the objective of examining the association between cyberbullying victimization and mental health problems and violent behaviors in American adolescents, comparisons are made between students who reported having been victims of cyberbullying and those who did not report it through: Bivariate Comparisons. Logistic Regression Model. Follow-up Period: No information. Losses: number / group: Mentions a general response rate of 60% for the 2015 YRBS.	among men. Weapon carrying significantly predicted cyberbullying victimization only among non-white students. All mental illness variables were significant for both white and non-white students.		

Garaigordobil 2020	Design: Descriptive and comparative cross-sectional study. Objectives: Analyze possible differences based on sexual orientation (heterosexual and non-heterosexual) in: The percentage of bullying and cyberbullying victims and aggressors. The amount of aggressive behavior suffered and performed in both groups. Compare the mental health of heterosexual and non-heterosexual adolescents who have been victims, aggressors, cyber-victims, and cyber-aggressors. Location and period: The study was carried out in the Basque Country, Spain.	Population: The study population is composed of adolescents from the Basque Country, Spain, aged between 13 and 17 years. The sample composition is: Size: 1,748 adolescents. Gender: 52.6% girls and 47.4% boys. Educational level: 60.2% in 3rd year of Secondary Education and 39.8% in 4th year. Type of school: 44.7% in public schools and 55.3% in private schools. Sexual orientation: 87.5% heterosexual and 12.5% non-heterosexual (0.7% gay, 0.2% lesbian, 5.7% bisexual, and 5.9% with undefined sexual orientation). Exposure: The exposure factor is the sexual orientation of adolescents. The research focuses on the comparison of two groups: Heterosexuals:	Number of subjects / group: The study analyzed a total sample of 1,748 adolescents divided into 2 groups according to their sexual orientation: Exposed group (non-heterosexuals): 12.5% of the total sample, equivalent to 219 adolescents. Non-exposed group (heterosexuals): 87.5% of the total sample, equivalent to 1,529 adolescents. Characteristics of the exposed cohort: Exposed individuals are defined as those adolescents who identify as non-heterosexual. This group represents 12.5% of the total sample, equivalent to 219 adolescents. The composition of the group in terms of their specific sexual orientation: Gay: 0.7% of the total sample. Lesbian: 0.2% of the total sample.	The study found that the percentage of bullying and cyberbullying victims was significantly higher in the group of non-heterosexual adolescents compared to heterosexual adolescents. Severe bullying victims: 9% of heterosexuals vs. 25.1% of non-heterosexuals. Severe cyber-victims: 6.2% of heterosexuals vs. 13.7% of non-heterosexuals. No significant differences were found in the percentage of aggressors and cyber-aggressors between the two sexual orientation groups. Non-heterosexual adolescents who were victims of bullying and cyberbullying reported having suffered a greater amount of aggressive behavior compared to heterosexual victims. Non-heterosexual	The percentage of victims and cyber-victims was significantly higher in the group of non-heterosexual adolescents, compared to heterosexual adolescents. Non-heterosexual victims and cyber-victims suffered a greater amount of aggressive bullying and cyberbullying behavior throughout their lives, compared to heterosexual victims. Non-heterosexual aggressors also showed a greater amount of in-person aggressive behavior than heterosexual aggressors. The study found no significant differences in the amount of cyberbullying carried out by heterosexual and non-heterosexual aggressors. Non-heterosexual victims, cyber-victims, aggressors, and	

		Adolescents who identify with a sexual orientation toward the opposite sex. Non-heterosexuals: Adolescents who do not identify exclusively with a sexual orientation toward the opposite sex, including adolescents who identify with different sexual orientations (Gay, Lesbian, Bisexual, Adolescents who are unsure of their sexual orientation). Clinical Effects: The study focuses on the mental health of adolescents and a variety of psychopathological symptoms, including: Beck Depression Inventory-II (BDI-II). Social Anxiety Scale for Adolescents (SAS-A). Symptom Checklist-90 Revised (SCL-90-R). Specific clinical effects analyzed in the study include: Depression. Social Anxiety. Somatization.	Bisexual: 5.7% of the total sample. Unsure of their sexual orientation: 5.9% of the total sample. Characteristics of the non-exposed cohort: The study defines the non-exposed group as the heterosexual adolescents who are part of the sample. These individuals are characterized by: Sexual orientation: They identify with a sexual orientation toward the opposite sex. Origin: They are students in the final cycle of Secondary Education in the Basque Country, Spain. Age: They are between 13 and 17 years old. Gender: The group is composed of 52.6% girls and 47.4% boys. Educational level: 60.2% of these adolescents are in 3rd year of Secondary Education and 39.8% are in 4th year. Type of school: 44.7%	aggressors showed a greater amount of in-person aggressive behavior compared to heterosexual aggressors. However, no differences were observed in the amount of cyberbullying. Compared to their heterosexual counterparts, non-heterosexual adolescents who were victims, cyber-victims, aggressors, or cyber-aggressors showed a worse state of mental health. Non-heterosexual victims and aggressors: Presented significantly higher levels of depression, social anxiety, and general psychopathological symptoms (somatization, obsession-compulsion, interpersonal sensitivity, etc.). Non-heterosexual cyber-victims and cyber-	cyber-aggressors presented significantly more symptoms of depression, social anxiety, and other psychopathological symptoms (somatization, obsession-compulsion, interpersonal sensitivity, etc.) than their heterosexual counterparts. The study highlights the need to develop interventions to reduce stigmatization and bullying/cyberbullying toward LGBT adolescents. The importance of family education in tolerance for diversity is emphasized, as well as school activities that promote respect for sexual diversity. The need to use the media to spread messages of tolerance and eliminate stereotypes and prejudices is suggested. The	

		Obsession-Compulsion. Interpersonal Sensitivity: Shyness, embarrassment, discomfort, and inhibition in interpersonal relationships. Anxiety. Hostility: Aggressive thoughts, feelings, and behaviors, anger, irritability, and resentment. Phobic Anxiety. Paranoid Ideation. Psychoticism.	attend public schools and 55.3% attend private schools. Exposure Factor: The exposure factor in this study is the sexual orientation of adolescents. The authors use it to divide the sample into two groups: Heterosexuals: Adolescents who identify with a sexual orientation toward the opposite sex. Non-heterosexuals: Adolescents who do not identify exclusively with a sexual orientation toward the opposite sex, including adolescents who identify with different sexual orientations (Gay, Lesbian, Bisexual, Adolescents who are unsure of their sexual orientation). Type of Comparison: The study performs a cross-sectional comparison between two groups of adolescents:	aggressors: Showed significantly higher levels of depression and psychopathological symptoms (except somatization in cyber-aggressors and obsession-compulsion in cyber-victims), but no differences were found in general social anxiety.	importance of clinical intervention to address the risk of suicide in people who suffer bullying/cyberbullying due to sexual orientation is mentioned.	

			heterosexual and non-heterosexual. Statistical Analysis: Chi-square tests are used to compare the prevalence of victimization and aggression between the groups. Analysis of variance (ANOVA and MANOVA) is performed to compare scores on mental health scales. Effect size is calculated to determine the magnitude of the differences found. Follow-up Period: No information. Losses: number / group: The study does not provide specific information about the number of participant losses throughout the research. It is not mentioned if there were students who dropped out of the study after giving their consent, nor are the reasons why this may have occurred specified. The lack of information about			

			participant losses is a limitation of the study, as this data is important for evaluating the validity of the results. Implications of Losses: Selection Bias: Participant losses can introduce selection bias if the characteristics of the students who dropped out of the study differ significantly from the characteristics of those who remained. Reduced Statistical Power: A high number of losses can reduce the statistical power of the study, making it difficult to detect significant differences between groups. Generalization of Results: The lack of information about losses makes it difficult to generalize the results to the general population of students in the final cycle of Secondary Education in the Basque Country.			

			Recommendations: In future research on bullying and cyberbullying, it is important for researchers to document and report the number of participant losses, as well as the reasons why they occurred. This information allows readers to evaluate the validity of the results and the generalization of the conclusions. Despite this limitation, the study provides valuable information on the prevalence of bullying and cyberbullying in adolescents in the Basque Country, and highlights the greater vulnerability of non-heterosexual students to this type of violence.			

Duarte 2018	Design: Cross-sectional study. Objectives: Main Objective: Understand the relationship between cyberbullying, minority status, and mental health in adolescents. Specific Objectives: Determine the prevalence of cyberbullying in adolescents. Examine the correlation between minority status and participation in cyberbullying. Evaluate the association between cyberbullying and mental health symptoms. Identify subgroups of adolescents at higher risk of suffering the negative mental health consequences of cyberbullying. Location and period: The study was conducted in an urban pediatric emergency department in the northeastern United	Population: The study population is defined as English-speaking adolescents, aged between 13 and 17, who came to the pediatric emergency service for any reason. Sample size: Of the 1,190 eligible adolescents, 1,063 consented and assented to participate in the screening survey, and 1,031 successfully completed the survey, representing a response rate of 86.6%. Exposure: The exposure factor is defined as participation in cyberbullying, either as a victim, perpetrator, or both. To measure exposure, the study uses two questions from the "Student School Survey." The levels of exposure to cyberbullying are established according to the frequency of experiences reported	Number of subjects / group: The study was conducted on a total of 1,031 adolescents. Characteristics of the exposed cohort: The study describes the characteristics of the exposed group: Gender: 57.3% of adolescents exposed to cyberbullying were female. Race: 35.4% of exposed adolescents identified as non-white. Ethnicity: 26.7% of exposed adolescents identified as Hispanic. Socioeconomic level: 53.8% of exposed adolescents were classified as low socioeconomic level. Sexual orientation: 13.2% of exposed adolescents identified as LGB (lesbian, gay, or bisexual). Age: The mean age of exposed adolescents was 14.8 years, with a standard deviation of 1.31 years. Social media use: Exposed adolescents used an	The study found a high prevalence of cyberbullying among adolescents attending a pediatric emergency department. 24.6% of participants reported at least one experience of cyberbullying in the last year. Minority sexual orientation (LGB) was significantly correlated with a higher probability of participating in cyberbullying. LGB adolescents were 2.49 times more likely to be involved in cyberbullying than their heterosexual peers, even after controlling for other demographic factors and social media use. Greater use of social media platforms was associated with a higher probability of participating in cyberbullying. Adolescents who used a greater number of social	The study found a high prevalence of cyberbullying among adolescents who came to the emergency department. This figure underscores the importance of addressing cyberbullying as a public health problem that affects a significant number of young people. Minority sexual orientation (LGB) was identified as a significant risk factor for participation in cyberbullying. The study confirmed that adolescents who participated in cyberbullying were more likely to experience negative mental health symptoms, such as suicidal ideation, depressive symptoms, and PTSD symptoms. The study found a correlation between greater use of technology (measured	

	States. Data collection was carried out for nine months, between February and December 2015.	by the participants: Victim. Perpetrator. Victim-perpetrator. Clinical Effects: The study focuses on three aspects of mental health that have been linked to cyberbullying: Suicidal ideation. Depressive symptoms. Post-Traumatic Stress Disorder symptoms.	average of 2.68 different social media platforms, with a standard deviation of 1.21. Characteristics of the non-exposed cohort: No information. Exposure Factor: empty. Type of Comparison: This is a cross-sectional study that compares the characteristics and mental health outcomes of two groups of adolescents in terms of: Demographic variables: gender, race, ethnicity, socioeconomic level, and sexual orientation. Social media use: number of platforms used. Mental health outcomes: suicidal ideation, depressive symptoms, and PTSD symptoms. Statistical Analysis: To evaluate the differences between the groups, statistical tests such as the chi-square test and logistic regression	media platforms were 1.16 times more likely to participate in cyberbullying, even after controlling for demographic factors. Adolescents who participated in cyberbullying had a higher prevalence of negative mental health symptoms, such as suicidal ideation, depressive symptoms, and PTSD symptoms, compared to adolescents who did not experience cyberbullying. Among adolescents who experienced cyberbullying, LGB sexual orientation was the only demographic factor that significantly correlated with a higher probability of reporting depressive and PTSD symptoms. Contrary to some previous studies, this study did not find a significant correlation between race, ethnicity, or gender and participation in	by the number of social media platforms used) and a greater likelihood of participation in cyberbullying.	

			were used. Follow-up Period: No information. Losses: number / group: Of the 1,190 eligible adolescents, 1,063 consented and assented to participate in the screening survey, and 1,031 successfully completed the survey, representing a response rate of 86.6%.	cyberbullying or negative mental health symptoms after adjusting for other factors. Low socioeconomic status (SES) correlated with a higher probability of participating in cyberbullying only after controlling for other demographic factors and social media use.		

Fajardo-Bullón 2021	Design: Cross-sectional, descriptive, and comparative study. Objectives: Compare total scores in mental health and cyberbullying (victims and perpetrators) in Spanish and Colombian adolescents. Analyze the relationship between mental health and having or not having a high-grade (severe) cyber-victim or cyber-aggressor profile due to the use of mobile phones and the Internet. Location and period: The study was conducted in two countries: Spain and Colombia. As for the period of realization, the questionnaires were administered during the 2014/2015 school year.	Population: The study population is composed of school-aged adolescents from Spain and Colombia. Spain: Students of Compulsory Secondary Education (ESO) from the Extremadura region, aged between 12 and 17 years. Colombia: Students aged between 11 and 17 years, from 5 schools in Bucaramanga and its metropolitan area. The study used a probabilistic cluster sampling, randomly selecting schools and groups in both locations. Exposure: The study defines the exposure factor as participation in cyberbullying, specifying different levels or profiles: Cyber-victim: A student who is the object of harassment, persecution, denigration, violation of privacy, or social exclusion through information and communication technologies, such as the internet or mobile phones. Cyber-aggressor: A student who carries out	Number of subjects / group: The number of subjects in each group are: Spain: Severe cyber-victims by mobile phone: 7.7% of the sample. Severe cyber-victims by internet: 5.4% of the sample. Severe cyber-aggressors: 6.1% of the sample. Colombia: Severe cyber-victims by mobile phone: 4.9% of the sample. Severe cyber-victims by internet: 6.8% of the sample. Severe cyber-aggressors: 9.6% of the sample. Characteristics of the exposed cohort: Exposed individuals refer to those classified as "severe cyber-victims" and "severe cyber-aggressors," based on their use of mobile phones and the internet. The "severe" classification is based on obtaining scores equal to or above the 95th percentile on the CYBVYC (victimization) and CYBAGRESS (aggression) scales. Characteristics of the non-exposed cohort: No information. Exposure Factor: The	Scores on the four SDQ scales and the total difficulties score were significantly higher in Colombian adolescents compared to Spanish adolescents. Colombian adolescents reported significantly higher levels of cyber-victimization via the Internet compared to Spanish adolescents. Colombian adolescents also obtained significantly higher scores in cyber-aggression than Spanish adolescents. Spanish adolescents reported significantly higher levels of cyber-victimization via mobile phone compared to Colombian adolescents. In both Spain and Colombia, adolescents who were classified as "severe cyber-victims" or "severe cyber-aggressors" obtained significantly higher scores on the SDQ scales and the total difficulties score compared to those who did not belong to these profiles. A positive and significant correlation	Differences exist between countries in mental health difficulties and cyberbullying. There is a great impact of severe cyberbullying on mental health. A positive correlation exists between mental health difficulties, cyber-victimization, and cyber-aggression.	

Chang 2013	Design: Cross-sectional cohort study. Objectives: Evaluate the prevalence of cyberbullying and school bullying. Examine the relationship between cyberbullying, school bullying, and mental health. Identify the characteristics of adolescents related to cyberbullying, school bullying, and psychological effects. Location and period: The study was conducted in 26 secondary schools in Taipei (Taiwan) during the year 2010.	Population: Study population: 10th-grade students in secondary schools in Taipei (Taiwan). A total of 2992 students from 102 classes in 26 secondary schools were included. 52% of the participants were male and 48% female. Exposure: The study examines the relationships between cyberbullying, school bullying, and mental health in Taiwanese adolescents, the main exposure factors would be cyberbullying and school bullying, specifying the different levels of participation. Clinical Effects: The study does examine two aspects of mental health that can be considered as indicators of psychological well-being and that, in severe cases, could be related to the appearance of clinical	Number of subjects / group: Cyberbullying: Exposed: Cyberbullying victims: 551. Cyber-aggressors: 174. Cyber-aggressor-victim: 336. Total exposed: 1061. Non-exposed: 1931. School Bullying: Exposed: School bullying victims: 244. School aggressors: 317. School aggressor-victim: 151. Total exposed: 712. Non-exposed: 2280. Characteristics of the exposed cohort: Variables such as: Sex. Academic Performance. Household Poverty. Risky Internet Behavior. Experiences of Cyberbullying and School Bullying. Characteristics of the non-exposed cohort: Variables such as: Sex. Academic Performance. Household Poverty. Risky Internet	More than a third of the students (35.4%) had participated in cyberbullying and/or had been victims of it in the last year. 18.4% of the students had been victims of cyberbullying. 5.8% of the students had bullied others online. 11.2% of the students had bullied others online and had also been victims of cyberbullying. A quarter of the students (23.9%) had participated in school bullying and/or had been victims of it in the last year. 8.2% of the students had been victims of school bullying. 10.6% of the students had bullied others at school. 5.1% of the students had bullied others at school and had also been victims of school bullying. Correlation between Cyberbullying and School Bullying: Students who had	Both cyberbullying and school bullying are frequent problems among Taiwanese adolescents. There is a strong correlation between cyberbullying and school bullying. Both cyberbullying and school bullying are associated with an increased risk of mental health problems, such as low self-esteem and depression. It is necessary to implement effective prevention and intervention programs to address cyberbullying and school bullying in schools.	

		problems: Self-Esteem: through the Rosenberg Self-Esteem Scale. Depression: using the Center for Epidemiological Studies Depression Scale (CES-D).	Behavior. Experiences of Cyberbullying and School Bullying. Exposure Factor: The study defines cyberbullying and school bullying using questionnaires with specific questions about the frequency of certain behaviors. The definition of exposure is based on the frequency of these behaviors. Cyberbullying. School bullying. Type of Comparison: The study performs several types of comparisons to analyze the relationship between cyberbullying, school bullying, and mental health in Taiwanese adolescents. The following methods are described as comparison: Comparison of the Prevalence of Cyberbullying and School Bullying, Comparison of the	been victims of cyberbullying were more likely to have also been victims of school bullying. Students who had bullied others online were more likely to have bullied others at school. Students who had been victims of cyberbullying and had bullied others online were more likely to have been victims and have bullied others at school. Risk Factors for Cyberbullying and School Bullying: Male students were more likely to participate in cyberbullying and school bullying. Students with low academic performance were more likely to be victims of cyberbullying and school bullying. Students living in poverty were more likely to be victims of school bullying. Students who engaged in risky		

			Experiences of Cyberbullying and School Bullying, Comparison of Mental Health between Different Groups, Multivariate analysis is performed. Follow-up Period: Study conducted in 2010. Losses: number / group: The response rate was 80%.	Internet behaviors (e.g., posting personal information, photos, or using a webcam to chat with strangers) were more likely to participate in cyberbullying and school bullying. Consequences of Cyberbullying and School Bullying for Mental Health: Victims of cyberbullying and school bullying were more likely to have low self-esteem and high levels of depression. Students who had bullied others online and had been victims of cyberbullying (cyber-victim-aggressors) had the lowest levels of self-esteem and the highest levels of depression. After controlling for sex, academic performance, and household poverty, victims of cyberbullying and school bullying, as well as aggressors		

				and cyber-victim-aggressors, were more likely to have high levels of depression. Most Frequent Types of Cyberbullying and School Bullying: The most frequent type of cyberbullying that students were victims of was unwanted sexual solicitation. The most frequent type of school bullying that students were victims of was social bullying (e.g., being excluded from groups).		

Caetano 2016	Design: Descriptive cross-sectional study. Objectives: Main objective: Characterize cyberbullying in a sample of Portuguese adolescents, analyzing the emotions experienced by both victims and aggressors. Secondary objectives: Identify and interpret the emotions experienced by young people involved in cyberbullying situations, either as victims or as aggressors. Relate the emotions experienced by victims and aggressors to sociodemographic variables such as sex, school level, school, and municipality. Location and period: The study was conducted in Portugal, covering the northern, central, and southern regions of the country. The period of the	Population: 3,525 students from 23 school clusters located in the northern, central, and southern regions of the country. Students in the 6th, 8th, and 11th year of schooling, aged between 10 and 23. A distribution of 1,683 male students (47.8%) and 1,837 female students (52.1%). Exposure: The subjects who have suffered cyberbullying could be understood as the exposure factor. Clinical Effects: No information.	Number of subjects / group: Subjects exposed to cyberbullying: Victims: A total of 267 students (7.6% of the sample) reported having been victims of cyberbullying during the last year. Aggressors: A total of 138 students (3.9% of the sample) admitted having been aggressors in cyberbullying situations during the last year. Characteristics of the exposed cohort: Students in basic and secondary education in Portugal, highlighting age and sex, distinguishing between victims and aggressors. Characteristics of the non-exposed cohort: No information. Exposure Factor: No information. Type of Comparison: The study performs several types of comparisons to	Regarding the victims' emotions, the results are: Sadness, desire for revenge, and fear are the most frequent emotions in victims. Humiliation and injustice are moral emotions that arise from the perception of transgression by the aggressor. Victims rarely report guilt, which contrasts with other types of victimization where self-blame is more common. Regarding the aggressors' emotions: Satisfaction, indifference, relief, and pleasure are the most reported emotions by aggressors. Although less frequent, some aggressors also report regret, guilt, anger with themselves, and sadness, indicating the presence of a moral judgment. Male victims report greater sadness, fear, insecurity, and desire	The study, based on a questionnaire applied to 3,525 Portuguese adolescents, reaches the following main conclusions: Victims mostly experience sadness, desire for revenge, and fear. Emotions associated with helplessness and lack of support are also observed, as well as the tendency to avoid social contact; they rarely report guilt. Aggressors most frequently present satisfaction, indifference, relief, and pleasure. These emotions could indicate a lack of empathy and a hedonistic and self-centered orientation. However, a significant percentage also reports moral emotions such as guilt, regret, and sadness. There is a notable discrepancy between the emotions that victims report and those that aggressors	

	study was between March and May 2012.		analyze the emotions associated with cyberbullying. Compares the emotions experienced by victims with the emotions that aggressors report feeling. Compares the emotions that victims express having felt with the emotions that aggressors believe the victims felt. Analyzes the relationship between the emotions experienced and variables such as sex, school level, school, and municipality. Spearman's correlation coefficients and chi-square tests are used to identify significant differences. Follow-up Period: No information. Losses: number / group: No information.	for revenge than female victims. Female aggressors show greater insecurity, relief, and confusion than male aggressors. Regret in aggressors and the feeling of terror, insecurity, and confusion in male victims vary significantly between schools, suggesting the influence of the school climate and cyberbullying prevention policies. Guilt and regret in aggressors also differ between municipalities.	attribute to them. Minimization of the seriousness of the actions and the attribution of one's own emotions to the victims are behaviors observed in aggressors. Male victims report greater sadness, fear, insecurity, and desire for revenge than female victims, while female aggressors show greater insecurity, relief, and confusion than male aggressors. Fun as an emotion is more frequent in aggressors with a higher school level. Significant differences are observed in the emotions experienced depending on the school and the municipality. This suggests the influence of the school ethos and local culture on the experience of cyberbullying. The study highlights the need to develop	

					socioemotional and ethical competencies to prevent and address cyberbullying. These competencies include empathy, emotional management, conflict resolution, and self-esteem. The importance of a systemic approach that involves students, parents, schools, and communities to create a positive school climate and a culture of cyberbullying prevention is underlined. The active participation of students in prevention policies and open dialogue with parents are crucial aspects in addressing the problem. It is necessary to break the cycle of violence and promote shared responsibility in the fight against cyberbullying.	

Smokowski, 2014	Design: Prospective cohort study. Objectives: Analyze the effects of bullying victimization over time and compare the results of different victim groups (past, current, and chronic). This approach goes beyond simply identifying an association between bullying and student difficulties; it seeks to understand the complexity of the bullying experience and its long-term consequences in a specific rural environment. Location and period: Multicenter study in two rural counties in the southeastern United States. Two years: 2011, 2012.	Population: The study population is characterized as a diverse group of rural adolescents, mostly from low socioeconomic status, with a significant representation of different racial and ethnic groups. Exposure: The main exposure factor is bullying victimization, which is divided into two types: physical/verbal bullying and cyberbullying. Clinical Effects: The study focuses on analyzing the impact of bullying victimization in three main areas of adolescent development in rural areas: school experiences, social support, and mental health.	Number of subjects / group: These participants were classified into different groups according to their experience of physical/verbal bullying and cyberbullying victimization. For physical/verbal bullying victimization: Non-victims: 2,157 (69%). Past victims: 376 (12%). Current victims: 250 (8%). Chronic victims: 344 (11%). For cyberbullying victimization: Non-victims: 2,658 (85%). Past victims: 219 (7%). Current victims: 156 (5%). Chronic victims: 94 (3%). Characteristics of the exposed cohort: The general composition of the sample is described; gender, racial diversity, socioeconomic level, family structure. Characteristics of the non-exposed cohort: This group is defined	The study results highlight the importance of addressing bullying in rural schools, as any type of victimization, whether physical/verbal, cyberbullying, past, current, or chronic, can have lasting negative consequences on young people's development.	The study provides solid evidence on the negative effects of bullying on rural adolescents. The findings underscore the need for coordinated action between schools, families, and communities to prevent and address bullying comprehensively and effectively.	

			as those students who did not report having been victims of physical/verbal bullying or cyberbullying at either of the two data collection times (Year 1 and Year 2). Exposure Factor: The sources generally define bullying, mentioning the five forms: physical, verbal, social, extortion, and cyber. The study focuses on two types of victimization: physical/verbal bullying and cyberbullying. Measurement of victimization: Two dichotomous (yes/no) questions are used in the "School Success Profile" (SSP) questionnaire. Type of Comparison: The study combines both types of comparison to analyze the relationship between bullying victimization and adolescent well-			

			being. By comparing "non-victims" with the different victim groups, the general impact of exposure to bullying is evaluated. The distinction between the three victim groups allows analysis of whether the chronicity of victimization exacerbates the negative effects on school experiences, social support, and mental health. Follow-up Period: The follow-up time is two years. Losses: number / group: No information.			

Dradas 2014	Design: Retrospective cohort study. Objectives: Focuses on the problem of school bullying and cyberbullying among Argentine adolescents, as well as its relationship with suicidal behaviors. Location and period: Argentina. Period: 2018.	Population: In Argentine students in grades 8 to 12. More than half of the participants were female. Exposure: The study clearly defines the exposure factor as harassment, specifying two distinct levels: traditional harassment and cyber-harassment. Clinical Effects: The study focuses on the relationship between victimization by school harassment (traditional and cyber) and suicidal behaviors. Specifically, two outcome variables are studied: suicidal ideation and suicide attempt.	Number of subjects / group: School Bullying: 26,750 students. Cyberbullying: 8,370 students. Characteristics of the exposed cohort: The study provides information on the age, sex, and school grade of the participants. Characteristics of the non-exposed cohort: The demographic characteristics of the non-exposed individuals are similar to those of the general sample. Exposure Factor: The sources provide a general definition of harassment and mention the questions used to measure exposure to traditional and cyber-harassment in the survey (GSHS). Type of Comparison: The study performs a group comparison to evaluate the association between school harassment	The study found a significant association between school harassment (traditional and cyber) and suicidal behaviors in Argentine adolescents. The results also suggest that connection with school, parents, and peers can play a protective role in mitigating the negative effects of harassment on mental health and suicide risk.	The study underlines the seriousness of school harassment and its impact on the mental health of Argentine adolescents. The findings emphasize the need for a comprehensive approach that addresses both harassment prevention and the promotion of resilience and well-being in young people.	

			victimization and suicidal behaviors. Logistic regression analysis is used and the PRR is calculated as a measure of association. Follow-up Period: 2018. Losses: number / group: The sources do not provide specific information about the number of losses for each group (exposed and non-exposed) in the study.			

Gomes 2024	Design: Mixed cross-sectional cohort study. Objectives: Analyze the emotions and emotional regulation strategies that adolescents adopt after observing various cyberbullying behaviors. Investigate the influence of the sex variable on the emotions felt by witnesses of cyberbullying and the emotional regulation strategies they adopt after observing four different cyberbullying scenarios. Investigate the role of school grade on the emotions and emotional regulation strategies of witnesses after observing these scenarios. Location and period: The study was conducted in a school in the Lisbon district (Portugal), from March 9, 2018, to June 8, 2018.	Population: Portuguese adolescents aged between 12 and 17 who attend a school in the Lisbon district. Exposure: The presence of different cyberbullying scenarios is defined as the exposure factor: Publication of a photograph of a girl in a bikini without her consent, with offensive comments. Publication of a direct threat to the victim. Publication of homophobic insults directed at the victim. Threat to share the victim's personal information without their permission to spread rumors. Clinical Effects: There are no clinical effects, but the negative effects of cyberbullying are studied: Impact on socio-affective development, academic performance, and general well-being. Anxiety, depression, and depressive symptoms. Difficulties in adaptively regulating emotions.	Number of subjects / group: The study had 101 students exposed to the cyberbullying scenarios through the Com@Viver game and 91 students in the control group, without specifying the distribution between the GC1 and GC2 subgroups. Characteristics of the exposed cohort: The exposed individuals in this study are the 101 students who were assigned to the experimental group. These students, aged between 12 and 17 and in the 7th or 8th grade, were exposed to four different cyberbullying scenarios within the Com@Viver game. The main characteristics of these exposed individuals are: Age: between 12 and 17 years old, with a mean of 13 years. Gender: 51.1% of the total sample were female. School Grade: 48.2% of the total sample were in 7th grade and 51.8% were in 8th grade. Nationality: all participants attend a school in the Lisbon	Both boys and girls showed greater emotional impact from photo publication, considering it a more serious form of aggression. Girls experienced more negative emotions than boys in general. Boys reported fewer negative emotions than girls in scenarios that did not involve photo publication, which could be due to a desensitization to this type of aggression. Adolescents mainly used strategies aimed at interpreting the emotional experience to understand the seriousness of the situation and be able to act. Rumination, in this context, was associated with greater empathy in spectators, contributing to prosocial behavior. Girls more frequently used the emotional regulation strategy of interpreting the emotional experience, through strategies such as situation recognition, situation selection (approach), and situation modification. Seventh	Both boys and girls consider the publication of the victim's photos as a more serious form of aggression, which provokes more intense emotional responses. Homophobic publications directed at the victim also trigger significant negative emotions, especially in girls. The study confirms the existence of gender differences in the way adolescents experience and regulate their emotions in the face of cyberbullying. Girls tend to experience negative emotions with greater intensity and more frequently use emotional regulation strategies oriented toward interpreting the emotional experience, seeking to understand the situation and the victim's perspective. Boys, for their part, report fewer negative emotions in some scenarios, which could indicate a greater desensitization to online aggression. Significant differences	

Baier 2018	Design: Cross-sectional cohort study. Objectives: Examine the relationship between school bullying and adolescent mental health in Germany. Location and period: Lower Saxony (Germany), during the spring of 2015.	Population: Ninth-grade students in the federal state of Lower Saxony, Germany. Average age: 14.9 years (SD = 0.73). Gender: 50.2% male. Migration background: 24.2%. Exposure: Exposure factor: school bullying, defined in different types: In-person bullying (physical and relational). Cyberbullying (psychological and sexual). Bullying by teachers (physical and psychological). Clinical Effects: The clinical effect is mental health through: Symptoms of depression and anxiety. Psychosomatic complaints.	Number of subjects / group: The exact number of students in each exposure group is not specified, but it can be deduced that a significant number of students reported having experienced different types of bullying. Characteristics of the exposed cohort: The study analyzes different levels of exposure to bullying, based on the frequency of experiences reported by students. Describing the following characteristics: Age. Gender. Migration background. School performance. Peer group relationships. Family relationships. Characteristics of the non-exposed cohort: No information. Exposure Factor: No information. Type of Comparison: The study examines the relationship between	Psychological cyberbullying is the factor that most influences the mental health of both boys and girls. Relational bullying, both by peers and teachers, also has a significant correlation with poor mental health. Sexual cyberbullying is related to mental health problems, but only in girls. Physical bullying by peers has a relatively low effect on mental health, especially in boys. The study found a contradictory, although small, effect of physical bullying by teachers. It is speculated that, unlike victims of peer bullying, students bullied by a teacher might receive sympathy from other students, which could improve their social status in the classroom. Social support correlates with better mental	Psychological cyberbullying is the factor that most influences the mental health of both boys and girls. Relational bullying, whether by peers or teachers, is also significantly correlated with more deteriorated mental health. Girls who suffer sexual cyberbullying present higher levels of depression, anxiety, and somatization. Physical bullying by peers has a relatively low effect on mental health, especially in boys. Social support correlates with better mental health, especially in girls. Negative family experiences are associated with worse mental health, regardless of gender. Low academic performance is related to mental health problems, particularly in girls.	

			continuous variables, i.e., the frequency of different types of bullying and the score on depression, anxiety, and psychosomatic symptom scales. Follow-up Period: No information. Losses: number / group: Initially, 672 classes were selected for the survey. However, some principals or teachers refused to participate, which reduced the sample to 545 classes with 12,650 enrolled students. Of the 12,650 students, 10,638 participated in the survey, resulting in a response rate of 68.5%. The reasons for non-participation were: Illness: 905 students. Lack of parental consent: 434 students. Refusal to participate: 255 students. Questionnaires with many missing values or joke responses: 51	health, especially in girls. Negative family experiences are associated with worse mental health, regardless of gender. Low grades are related to mental health problems, especially in girls. The study found almost identical correlations for both mental health measures (depression/anxiety and somatization).		

			students. Other reasons (such as pending tasks or school events): 367 students.			

Kim 2019	Design: Retrospective cross-sectional cohort study. Objectives: Examine sex differences in the relationship between cyberbullying victimization and various outcomes in adolescents. Understand if cyberbullying victimization affects male and female adolescents differently in terms of these outcomes. Location and period: Ontario (Canada) during the year 2013.	Population: 4940 adolescents in Ontario in grades 7 to 12 in 2013. Exposure: Cyberbullying victimization, through 3 levels: Level 0: never. Level 1: once. Level 2: 2 or more times. Clinical Effects: In the field of Mental Health: Psychological Distress: Evaluated using a 10-item checklist that measures the frequency with which anxiety and depression are experienced in the last 4 weeks. Response options, coded from 1 ("none of the time") to 5 ("all the time"), are summed to generate a total score. A binary measure of psychological distress is created, where "0" represents low to moderate distress (scores...	Number of subjects / group: subjects exposed and not exposed to cyberbullying victimization: Women: Not exposed: 3827 (77.3% of women). Exposed once: 465 (9.4% of women). Exposed two or more times: 658 (13.3% of women). Men: Not exposed: 1991 (83.9% of men). Exposed once: 410 (8.3% of men). Exposed two or more times: 390 (7.8% of men). In total, the sample included: Not exposed: 5818 (80.2% of the total sample). Exposed at least once: 1465 (19.8% of the total sample). The study excluded 263 participants (5.3% of the original sample) who reported not using the internet. These participants were classified as not exposed to cyberbullying	The prevalence of cyberbullying victimization in the last year was 19.8% in the total sample of adolescents. 9.4% of women were cyberbullied once, and 13.3% two or more times. 8.3% of men were cyberbullied once, and 7.8% two or more times. Cyberbullying victimization was associated with an increased likelihood of experiencing mental health problems, including suicidal ideation, psychological distress, and delinquency. Cyberbullying victimization was significantly associated with substance use only in women.	Adolescents exposed to cyberbullying victimization have a higher probability of presenting poorer mental health outcomes, substance use outcomes, and suicidal ideation. The study reveals a greater risk among female adolescents compared to male adolescents.	

			victimization. Characteristics of the exposed cohort: No information. Characteristics of the non-exposed cohort: No information. Exposure Factor: Not exposed (0): Adolescents who answered "I don't use the internet" or "never" were considered not exposed. Exposed once (1): Adolescents who reported a single instance of cyberbullying. Exposed two or more times (2): Adolescents who reported two or more instances of victimization. Type of Comparison: The study performs a comparison between sexes to analyze the differences in the association between cyberbullying victimization and various mental health variables, suicidal ideation, and substance use in			

			adolescents. Follow-up Period: No information. Losses: number / group: No information.			

Calpbinici 2019	Design: Descriptive cross-sectional study. Objectives: Investigate the relationship between adolescents' virtual behaviors, such as Internet and mobile phone use, cyberbullying, and their perceived mental health. Location and period: The study was carried out in Nevşehir (Turkey), with high school students. during the 2013-2014 academic year.	Population: 8,311 high school students, including 4,199 male and 4,112 female students, attending public and private high schools in the city of Nevşehir, Turkey, during the 2013-2014 academic year. Exposure: Virtual behaviors can be considered as: Daily duration of internet use. Purpose of internet use. Place of internet use. Mobile phone use. Cyberbullying. Clinical Effects: The clinical effects under study are represented by the scores obtained on the five BSI subscales: Anxiety: evaluates the presence of symptoms such as nervousness, tension, tremors, excessive worry, and difficulty relaxing. Depression: measures the presence of symptoms such as sadness, pessimism, loss of interest in pleasant activities, fatigue, difficulty concentrating, and thoughts of death or suicide. Negative Self-Esteem: explores feelings of [illegible]	Number of subjects / group: The study does not clearly define exposure, but it can be determined that: Daily Duration of Internet Use: Exposed (use >1 hour). Non-exposed (use ≤1 hour and none). Cyberbullying: Exposed (performing cyberbullying). Non-exposed (not performing cyberbullying). Exposed (being exposed to cyberbullying). Non-exposed (not being exposed to cyberbullying). Mobile phone use for social media: Exposed (using for social media). Non-exposed (not using for social media). Characteristics of the exposed cohort: No. Characteristics of the non-exposed cohort: No. Exposure Factor: Partially. Type of Comparison: The study performs cross-sectional comparisons to evaluate the relationship between adolescents' virtual behaviors and their self-perceived mental	Almost half of the adolescents (49%) reported having participated in cyberbullying behaviors. More than half of the adolescents (61%) indicated having been victims of cyberbullying. Adolescents who used the Internet for more than one hour a day presented higher mean scores on the BSI subscales of anxiety, depression, low self-esteem, somatization, and hostility, compared to those who did not use the Internet or used it for one hour or less a day. Adolescents who did not use the Internet for studying showed higher mean scores on all BSI subscales, compared to those who did use it for studying. Adolescents who used the Internet for social media (Facebook/Skype/Twitter, etc.) obtained higher mean scores on all BSI subscales, compared to those who did not use it for this purpose. Adolescents who used the Internet to [illegible]	Adolescents who spend more than one hour a day using the internet present higher mean scores on the BSI subscales of anxiety, depression, low self-esteem, somatization, and hostility compared to those who do not use the internet or use it for one hour or less a day. The use of the internet for social media and games is associated with higher mean scores on all BSI subscales. Adolescents who have their own mobile phone show higher mean scores on the BSI subscale of hostility. The use of the mobile phone for social media is also related to higher mean scores on all BSI subscales. Adolescents who engage in cyberbullying or who are victims of it obtain higher mean scores on all BSI subscales, suggesting a negative impact on their mental health. The study authors highlight the importance for school nurses to raise adolescents' [illegible]	

Kim 2017	Design: Cross-sectional study. Objectives: Examine whether sex modifies the strength of the association between cyberbullying victimization and adolescents' emotional and behavioral problems. Compare the magnitude of the associations between adolescents' emotional and behavioral problems and cyberbullying victimization versus traditional forms of school bullying (social, verbal, and physical). Location and period: The study was carried out in the province of Ontario (Canada) between December 2014 and May 2015.	Population: A total of 31,124 students from grades 6 to 12 (equivalent to secondary education in some countries) in the province of Ontario (Canada) participated. Exposure: The study defines the exposure factor as school bullying victimization at least once or twice a month or more in the last 6 months, including both traditional forms and cyberbullying. Four types of victimization are specified: Physical bullying: Includes actions such as hitting, kicking, pushing, or damaging the victim's belongings. Verbal bullying: Refers to the use of words to hurt or humiliate the victim, such as insults, nicknames, threats, or teasing. Social bullying: Consists of isolating or excluding the victim from the	Number of subjects / group: Cyberbullying: Exposed: 2761 students (9.1% of the total sample). Non-exposed: It can be inferred that the rest of the sample, i.e., 28,363 students (90.9%), do not meet the cyberbullying exposure criterion defined in the study. Verbal bullying: Exposed: 5196 students (25.8% of the total sample). Non-exposed: 25,275 students (74.2%). Social bullying: Exposed: 6637 students (21.9% of the total sample). Non-exposed: 23,834 students (78.1%). Physical bullying: Exposed: 2854 students (9.5% of the total sample). Non-exposed: 28,267 students (90.5%). No type of bullying: Characteristics of the exposed cohort: Cyberbullying: Sex. Age. Ethnicity.	Cyberbullying was significantly associated with both emotional and behavioral problems, even after controlling for age and other forms of bullying. For women, the association between cyberbullying and emotional problems was stronger than for men. For men, the association between cyberbullying and behavioral problems was stronger than for women. Cyberbullying uniquely contributed to adolescents' emotional and behavioral problems, over and above traditional forms of bullying. Cyberbullying victimization was more prevalent in women than in men. Men reported more physical bullying than women. Women may be more vulnerable to emotional problems as a result of	Cyberbullying has a negative impact on adolescents' mental health. Cyberbullying uniquely contributes to mental health problems, beyond the effects of traditional bullying. The adolescent's sex influences the relationship between cyberbullying and mental health problems. Women are more likely to experience emotional problems as a result of cyberbullying. Men are more likely to develop behavioral problems after being victims of cyberbullying. The magnitude of the impact of cyberbullying is comparable, and in some cases greater, than that of traditional bullying.	

		peer group, spreading rumors, or manipulating others to reject them. Cyberbullying: Defined as intentional and repeated aggression carried out in an electronic context where there is a power imbalance between the aggressor and the victim. Clinical Effects: Emotional problems: The study uses 9 items from the Emotional Problems Scale (depression and anxiety). Behavioral problems: Measured using 13 items from the Conduct Problems Scale (conduct disorder and oppositional defiant disorder). Three additional items on conduct disorder were added: "(1) I use weapons when I fight, (2) I steal things from places other than home, and (3) I have broken into someone else's house."	Parents' educational level. Verbal bullying: Sex. Age. Ethnicity. Parents' educational level. Social bullying: Sex. Age. Ethnicity. Parents' educational level. Physical bullying: Sex. Age. Ethnicity. Parents' educational level. Characteristics of the non-exposed cohort: For non-exposed subjects, the following are determined: Sex. Age. Ethnicity. Parents' educational level. Exposure Factor: The study considers those who reported having experienced a type of bullying "once or twice a month or more" in the last 6 months as exposed. Type of Comparison: empty. Follow-up Period: empty. Losses: number / group: empty.	cyberbullying due to greater susceptibility to interpersonal stress and the tendency to ruminate on relational aggression experiences. For women, cyberbullying had a stronger association with emotional problems than verbal and physical bullying. For men, the effect of cyberbullying on emotional problems was greater than that of physical bullying. For both sexes, cyberbullying had significantly stronger associations with conduct problems than other forms of bullying.		

Kim 2017	Design: Cross-sectional study. Objectives: Examine whether sex modifies the strength of the association between cyberbullying victimization and adolescents' emotional and behavioral problems. Compare the magnitude of the associations between adolescents' emotional and behavioral problems and cyberbullying victimization versus traditional forms of school bullying (social, verbal, and physical). Location and period: The study was carried out in the province of Ontario (Canada) between December 2014 and May 2015.	Population: A total of 31,124 students from grades 6 to 12 (equivalent to secondary education in some countries) in the province of Ontario (Canada) participated. Exposure: The study defines the exposure factor as school bullying victimization at least once or twice a month or more in the last 6 months, including both traditional forms and cyberbullying. Four types of victimization are specified: Physical bullying: Includes actions such as hitting, kicking, pushing, or damaging the victim's belongings. Verbal bullying: Refers to the use of words to hurt or humiliate the victim, such as insults, nicknames, threats, or teasing. Social bullying: Consists of isolating or excluding the victim from the	Number of subjects / group: Cyberbullying: Exposed: 2761 students (9.1% of the total sample). Non-exposed: It can be inferred that the rest of the sample, i.e., 28,363 students (90.9%), do not meet the cyberbullying exposure criterion defined in the study. Verbal bullying: Exposed: 5196 students (25.8% of the total sample). Non-exposed: 25,275 students (74.2%). Social bullying: Exposed: 6637 students (21.9% of the total sample). Non-exposed: 23,834 students (78.1%). Physical bullying: Exposed: 2854 students (9.5% of the total sample). Non-exposed: 28,267 students (90.5%). No type of bullying: Characteristics of the exposed cohort: Cyberbullying: Sex. Age. Ethnicity.	Cyberbullying was significantly associated with both emotional and behavioral problems, even after controlling for age and other forms of bullying. For women, the association between cyberbullying and emotional problems was stronger than for men. For men, the association between cyberbullying and behavioral problems was stronger than for women. Cyberbullying uniquely contributed to adolescents' emotional and behavioral problems, over and above traditional forms of bullying. Cyberbullying victimization was more prevalent in women than in men. Men reported more physical bullying than women. Women may be more vulnerable to emotional problems as a result of	Cyberbullying has a negative impact on adolescents' mental health...	

		peer group, spreading rumors, or manipulating others to reject them. Cyberbullying: Defined as intentional and repeated aggression carried out in an electronic context where there is a power imbalance between the aggressor and the victim. Clinical Effects: Emotional problems: The study uses 9 items from the Emotional Problems Scale (depression and anxiety). Behavioral problems: Measured using 13 items from the Conduct Problems Scale (conduct disorder and oppositional defiant disorder). Three additional items on conduct disorder were added: "(1) I use weapons when I fight, (2) I steal things from places other than home, and (3) I have broken into someone else's house."	Parents' educational level. Verbal bullying: Sex. Age. Ethnicity. Parents' educational level. Social bullying: Sex. Age. Ethnicity. Parents' educational level. Physical bullying: Sex. Age. Ethnicity. Parents' educational level. Characteristics of the non-exposed cohort: For non-exposed subjects, the following are determined: Sex. Age. Ethnicity. Parents' educational level. Exposure Factor: The study considers those who reported having experienced a type of bullying "once or twice a month or more" in the last 6 months as exposed. Type of Comparison: empty. Follow-up Period: empty. Losses: number / group: empty.	cyberbullying due to greater susceptibility to interpersonal stress and the tendency to ruminate on relational aggression experiences. For women, cyberbullying had a stronger association with emotional problems than verbal and physical bullying. For men, the effect of cyberbullying on emotional problems was greater than that of physical bullying. For both sexes, cyberbullying had significantly stronger associations with conduct problems than other forms of bullying.		
