# Supplementary figures and images for: Study of the Relationship Between Cyberbullying and Mental Health in Adolescents—A Systematic Review
Source: Children (Basel). 2026 Mar 4;13(3):367. doi: 10.3390/children13030367 (PMC13025276; doi:10.3390/children13030367)

## Identification of studies via databases and registers

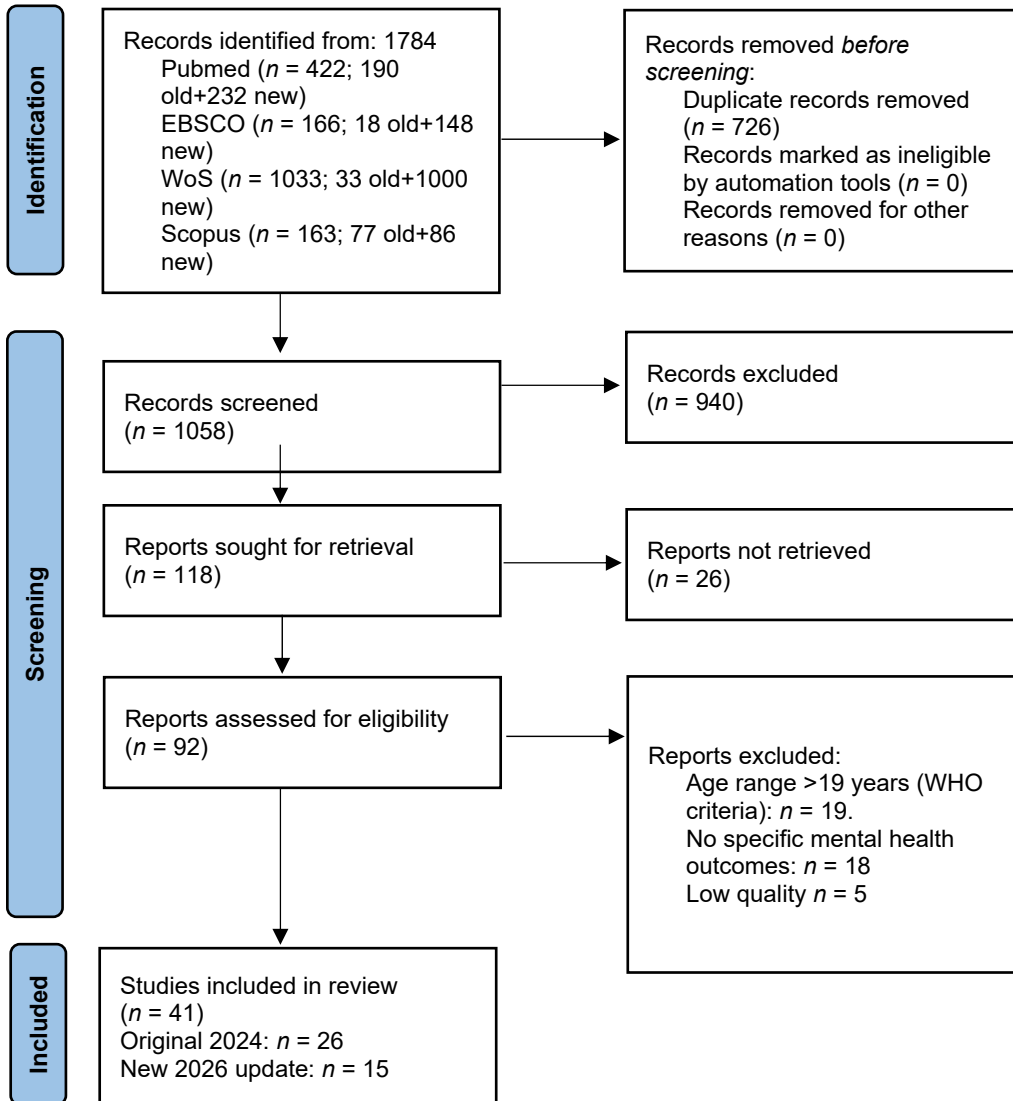

Supplement: Supplementary file 1 [file children-13-00367-s001.zip › PRISMA_2020_flow_diagram_new_SRs_v2.pdf]
